# Supplementary material for: Impact of tumor-parenchyma biomechanics on liver metastatic progression: a multi-model approach
Source: Sci Rep. 2021 Jan 18;11:1710. doi: 10.1038/s41598-020-78780-7 (PMC7813881; doi:10.1038/s41598-020-78780-7)
Supplement: Supplementary file 1 — Supplementary Information 1. [file 41598_2020_78780_MOESM1_ESM.pdf]

SUPPLEMENTARY MATERIAL FOR:
Impact of tumor-parenchyma biomechanics on liver metastatic progression: a multi-model approach

Yafei Wang[1,+] Erik Brodin[2,+] Kenichiro Nishii[2] Hermann B. Frieboes[3,4,5] Shannon Mumenthaler[6] Jessica L. Sparks[2,*] Paul Macklin[1,*]

[1]Department of Intelligent Systems Engineering, Indiana University. Bloomington, IN USA
[2]Department of Chemical, Paper and Biomedical Engineering, Miami University. Oxford, OH USA
[3]Department of Bioengineering, University of Louisville. Louisville, KY USA
[4]James Graham Brown Cancer Center, University of Louisville. Louisville, KY USA
[5]Center for Predictive Medicine, University of Louisville. Louisville, KY USA
[6]Lawrence J. Ellison Institute for Transformative Medicine, University of Southern California. Los Angeles, CA USA

[+]these authors contributed equally to this work
[*]co-corresponding authors: sparksj6@MiamiOH.edu (poroviscoelastic model) and macklinp@iu.edu (agent-based model and overall study)

Additional Figures

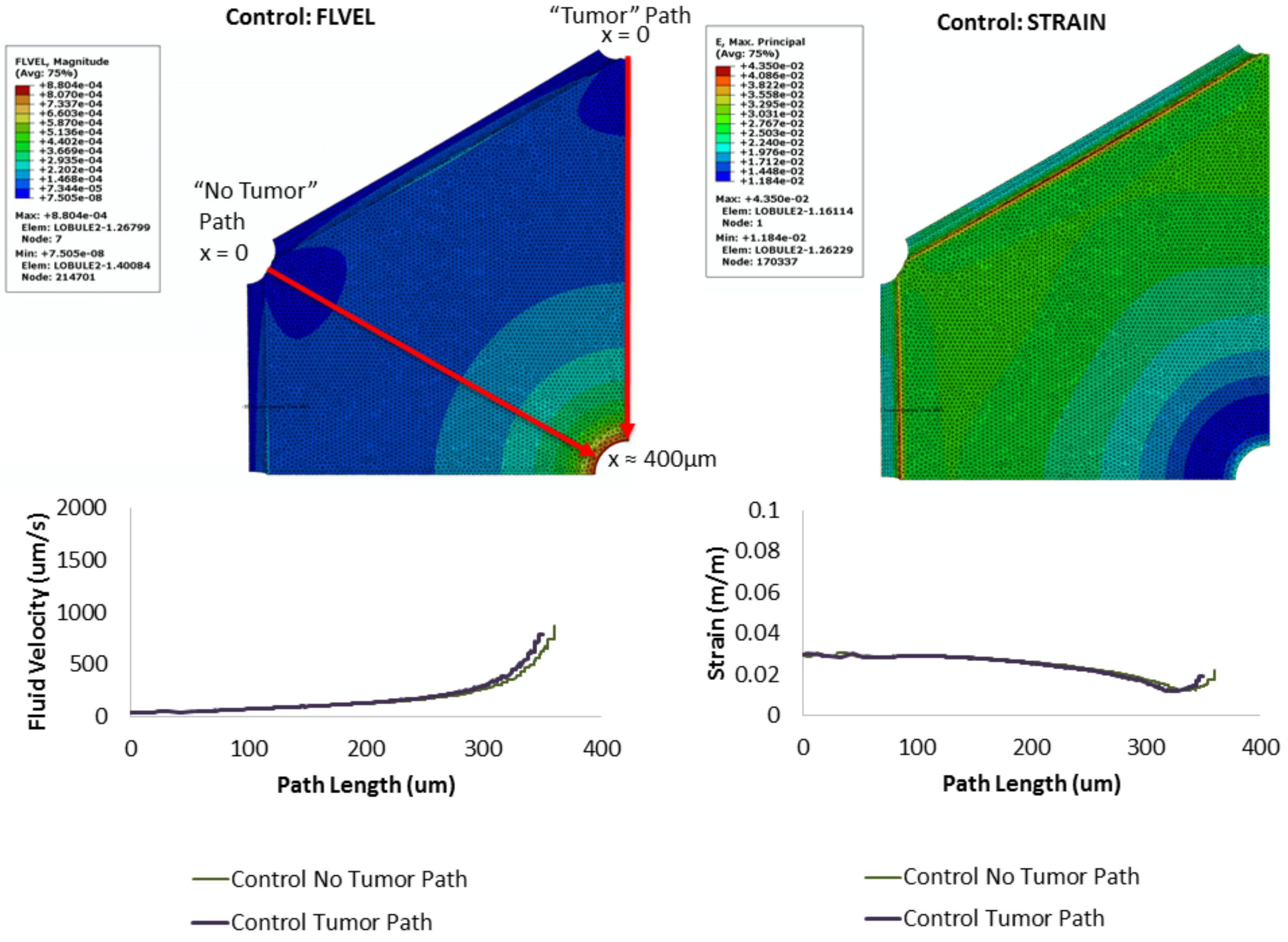

Figure S1. Hepatic flow: no tumor. The fluid velocity (left plots) and strain (right plots) are plotted across the lobule (top plots) and along "tumor" and "no tumor" paths (bottom plots). Flow accelerates as fluid approaches the central vein, whereas strain slightly decreases.

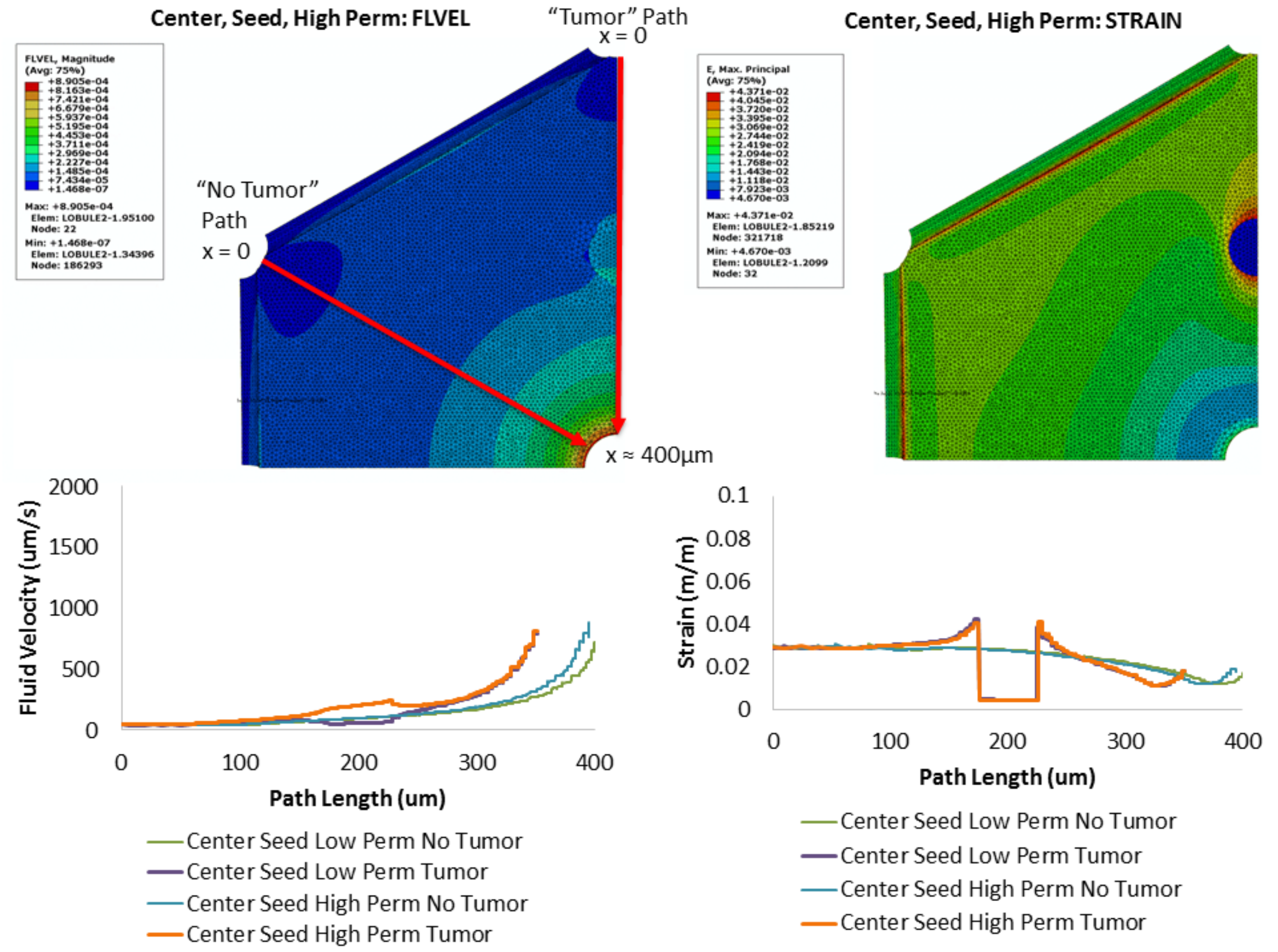

Figure S2. **Hepatic flow: small tumor seeds.** A 50 μm micrometastasis is seeded between the portal triad and central vein, with neutral pressure difference at the tumor boundary. The fluid velocity and strain along the non-tumor path are largely unaffected by the nearby tumor, whereas the fluid velocity increases slightly in highly permeable tumors, and decreases in less permeable metastatic seeds (left plots). The tissue strain is very low within the tumor but increases in the parenchyma immediately surrounding the tumor seed (right plots).

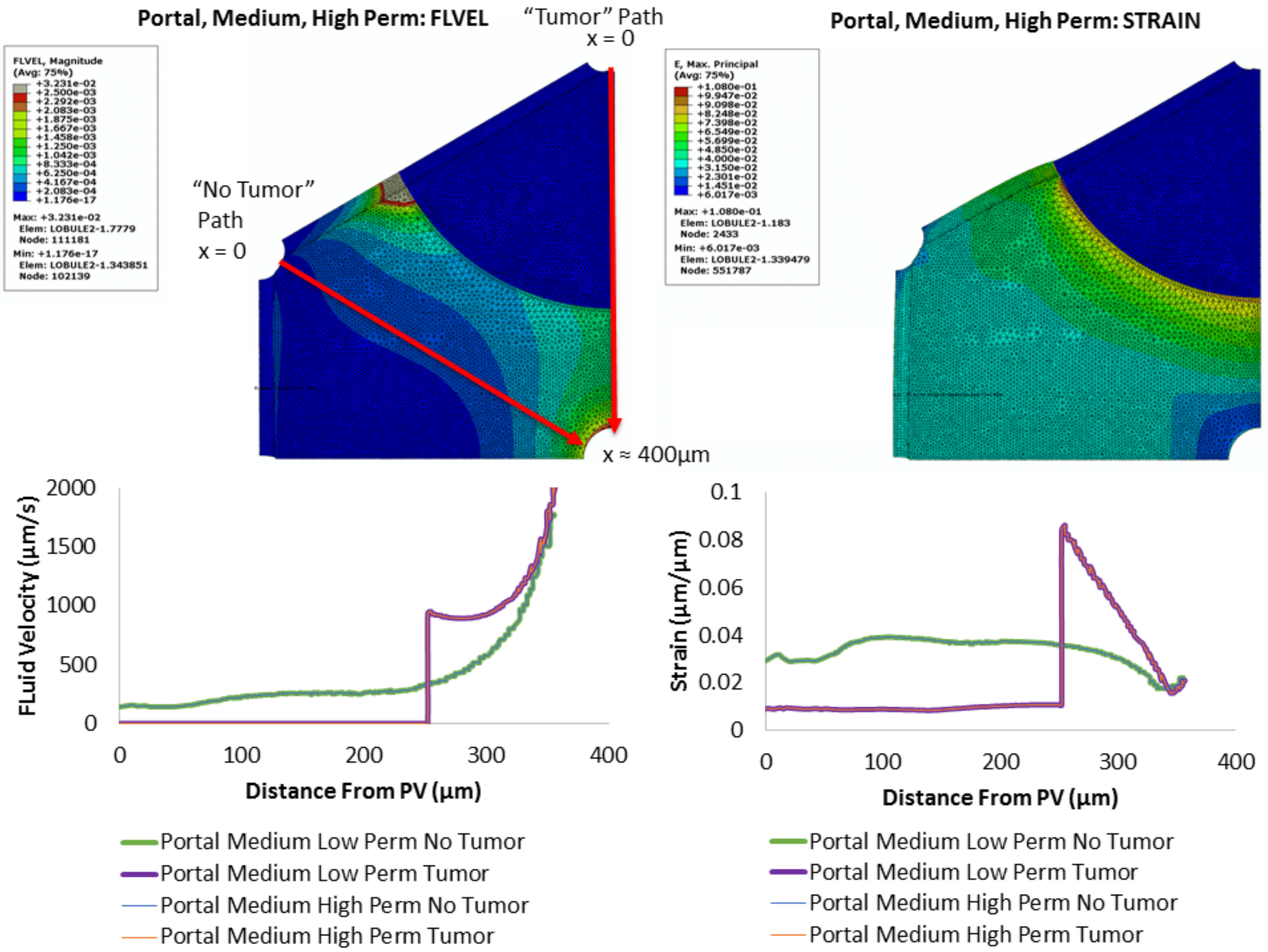

Figure S3. Hepatic flow: medium micrometastasis. A 400-μm micrometastasis disrupts a hepatic lobule near a portal triad, with tumor treated as a net pressure source. The fluid velocity increases in the parenchyma near the tumor but abruptly drops to zero inside the tumor. There is significant tissue strain in the parenchyma that peaks at the tumor edge and extends significantly into the surrounding tissue before dissipating. These results hold for both high and low tumor tissue permeability, and for neutral tumor pressure (results in supplementary materials.

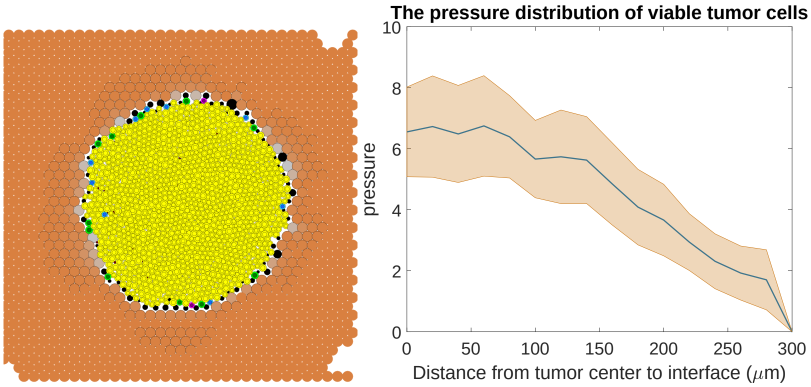

Figure S4. **Micrometastases without a mechanobiologic growth feedback.** *Left:* After 10 days of growth, almost all tumor cells have pressure exceeding 1.0, with considerable (non-physical) cell overlap. *Right:* We plot the mean (central curve) and standard deviation (shaded region) of the cell pressure versus distance from the tumor center of mass. Pressure peaks at ~ 7 nearest to the tumor center, gradually decreasing towards zero on the tumor edge where liver parenchyma apoptosis locally relieves the pressure. **Simulation parameters:** r_{E}=0.1 (1/min), r_{P}=0.01 (1/min) and d_{max}=1 μm.

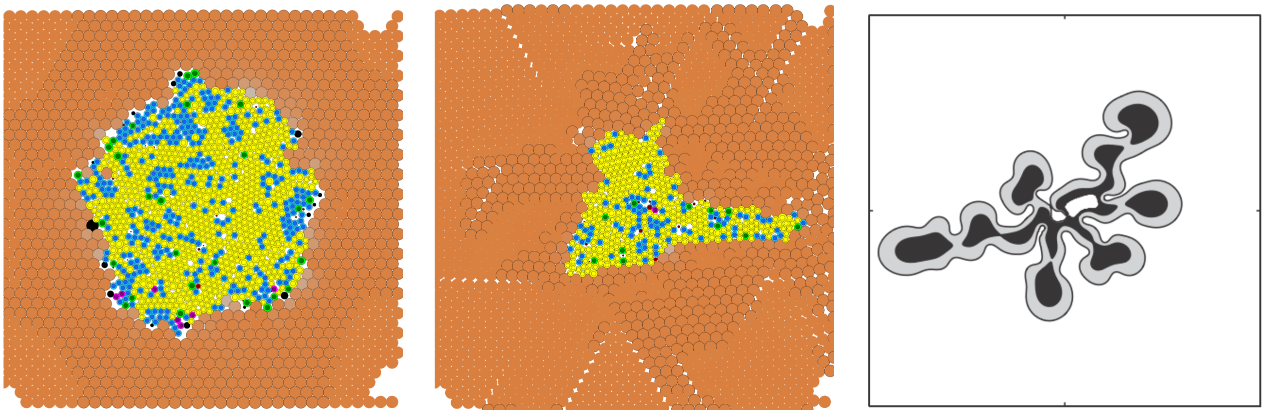

(a) r_{E}=0.1, r_{P}=0.01, d_{max}=1

(b) r_{E}=0.1, r_{P}=0.01, d_{max}=3

(c) continuum methods [1]

Figure S5. **Fingering metastatic growth: (a)-(b):** Growth into initially compliant parenchyma that tolerates deformation can lead to fingering morphologic instabilities, where tumor growth invades along directions of mechanical least resistance. Cells are colored as described in *Fig. 5* of main paper. **(c):** Macklin and Lowengrub previously used continuum methods to predict that tumor growth into relatively stiff, well-perfused tissue can lead to fingering-shaped morphological instabilities [1]. See *Fig. 8* of main paper for an example of CRC growth along paths of mechanical least resistance (yellow highlighted regions in the blue inset box).

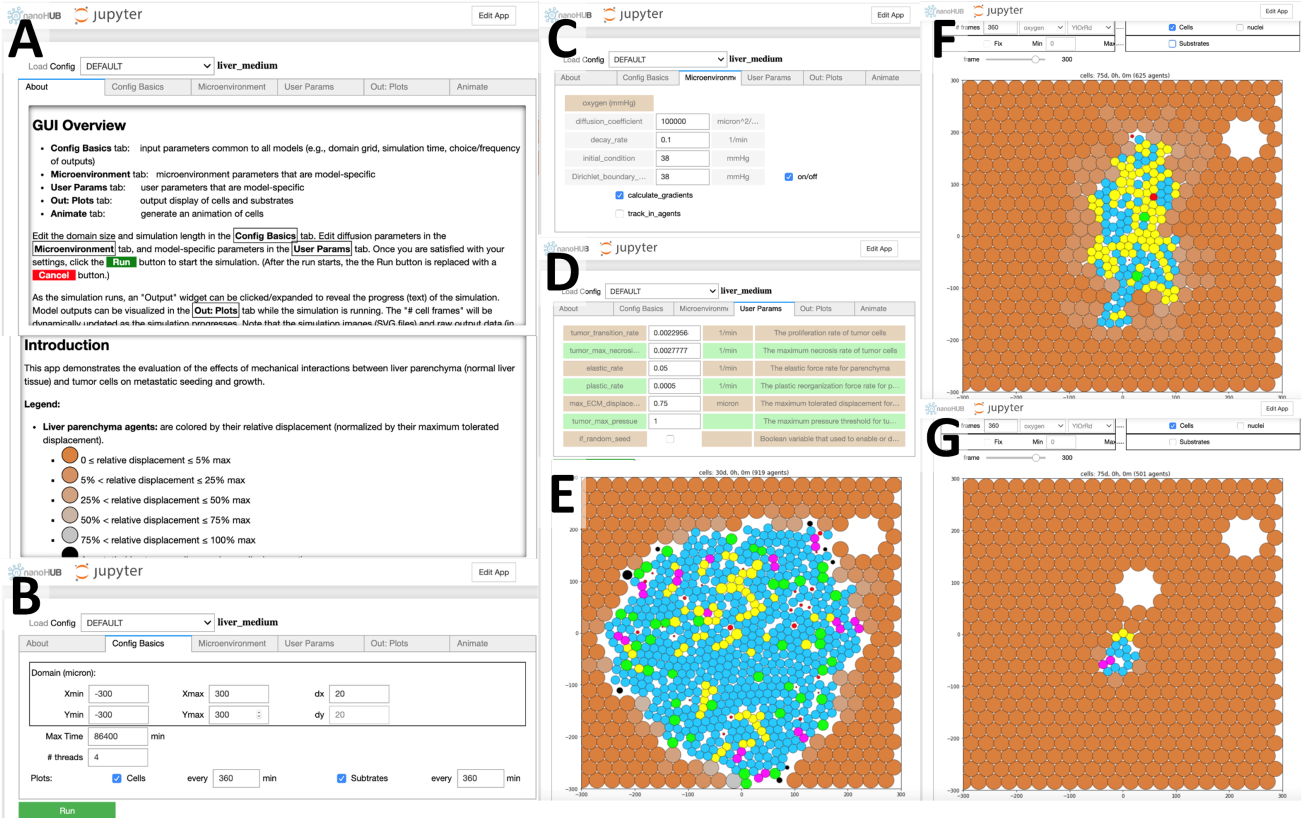

Figure S6. **Cloud-hosted interactive model:** Readers can interactively explore the agent-based model at https://nanohub.org/tools/pc4livermedium. (**A**) The *About* tab introduces the model, describes the plot legends, and gives run instructions. Users can modulate the domain size (**B**), diffusion parameters (**C**), and model-specific parameters (**D**). Simulation results can be viewed live in the *Out: Plots* tab, or animated as an mp4 movie in the *Animate* tab. Sample outputs are shown in **E**-**G**.

Supplementary Details on Results

Analysis of flow within micrometasases

Let us approximate fluid flow as identical to control conditions outside micrometastases and vanishingly small within tumors.

Using standard conservation of mass laws, we model the transport of a single growth substrate σ (e.g., oxygen or glucose) in liver tissue as

$$\frac{\partial \sigma}{\partial t} = -\nabla \cdot \vec{J} - \lambda \sigma \quad (1)$$

$$\vec{J} = -D \nabla \sigma + \sigma \vec{u}, \quad (2)$$

where $\vec{u}$ is the fluid flow field (from the PVE model), λ is the growth substrate consumption rate, D is diffusion coefficient, and with boundary conditions to be described further below.

Based on the typical fluid velocities in unobstructed liver ($||\vec{u}|| \sim 10^{-4}$ m/sec) and tumor region ($||\vec{u}|| \sim 0$ m/sec), we find (after nondimensionalization) that growth substrate biotransport is largely advective in the parenchyma and diffusive within tumor regions. See Appendix for expanded detail.

An analytical approximation to oxygen distribution in unobstructed parenchyma

We can further build on these results to obtain an approximate analytical solution to the quasi-steady substrate distribution within an unobstructed lobule. Because the flow is largely advective-reactive, we can simplify the original equation to

$$\frac{\partial \sigma}{\partial t} = -\nabla \cdot (\sigma \vec{u}) - \lambda \sigma. \quad (3)$$

Next, assuming incompressible flow, approximating flow as radially symmetric ($r = 0$ corresponds to the central vein), and matching to the spatial lobule size from the PVE model results, we can obtain an approximate analytical solution for biotransport for oxygen (σ) throughout unobstructed lobules:

$$u(r) = 6 \times 10^4 e^{-0.0075r} \quad (4)$$

$$\sigma(r) = 38.9 e^{0.0223\left(e^{0.0075r} - 1\right)}. \quad (5)$$

See Appendix for expanded mathematical details.

We plot the radial flow and oxygenation profiles for these parameters in a hepatic lobule with radius $R = 400\,\mu$m in Fig. S7; note that $\sigma(R) \approx 60$ mmHg, comparable to the portal venule oxygenation reported in [2].

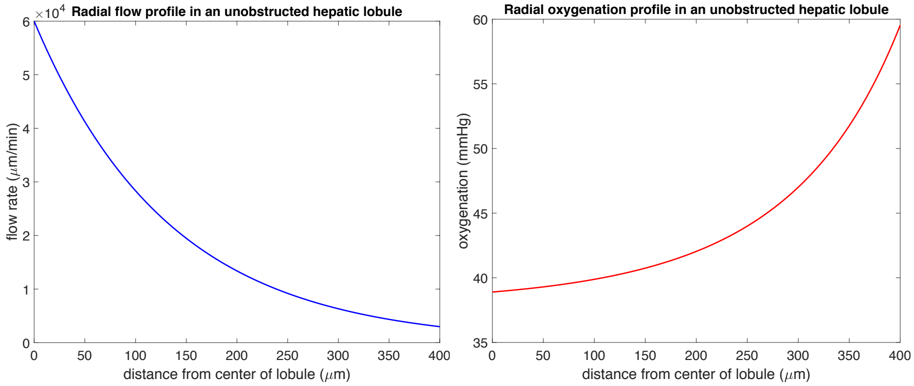

Figure S7. Approximate analytical solution to flow (**a**) and oxygen distribution (**b**) in an individual, unobstructed lobule

Approximate oxygen distribution in large tissue sections

We can extend the analytical single-lobule approximation to approximate advection-dominated perfusion in a large tissue section. We generated a large 1 cm^2 section of liver tissue with hundreds of hepatic lobules with central veins at positions $\{\vec{x}_i\}_{i=1}^n$. To approximate the quasi-steady oxygenation pO_2 at any position $\vec{x}$ of unobstructed tissue, let us define

$$r(\vec{x}) = \min\{||\vec{x}_i - \vec{x}||\}_{i=1}^n \quad (6)$$

to be the distance between $\vec{x}$ and the nearest central vein. Then we approximate

$$pO_2(\vec{x}) = \sigma\left(r(\vec{x})\right). \quad (7)$$

Here, σ is the radial oxygenation profile defined in Equation 5.

Note that this is equivalent to partitioning the 2-D tissue cross-section into a Voronoi mesh (with portal triads at the vertices (representing the flow source) and central veins in the centers) and assuming radial flow within each Voronoi polygon. We show this approximation for the 1 cm^2 tisue in Fig. S8.

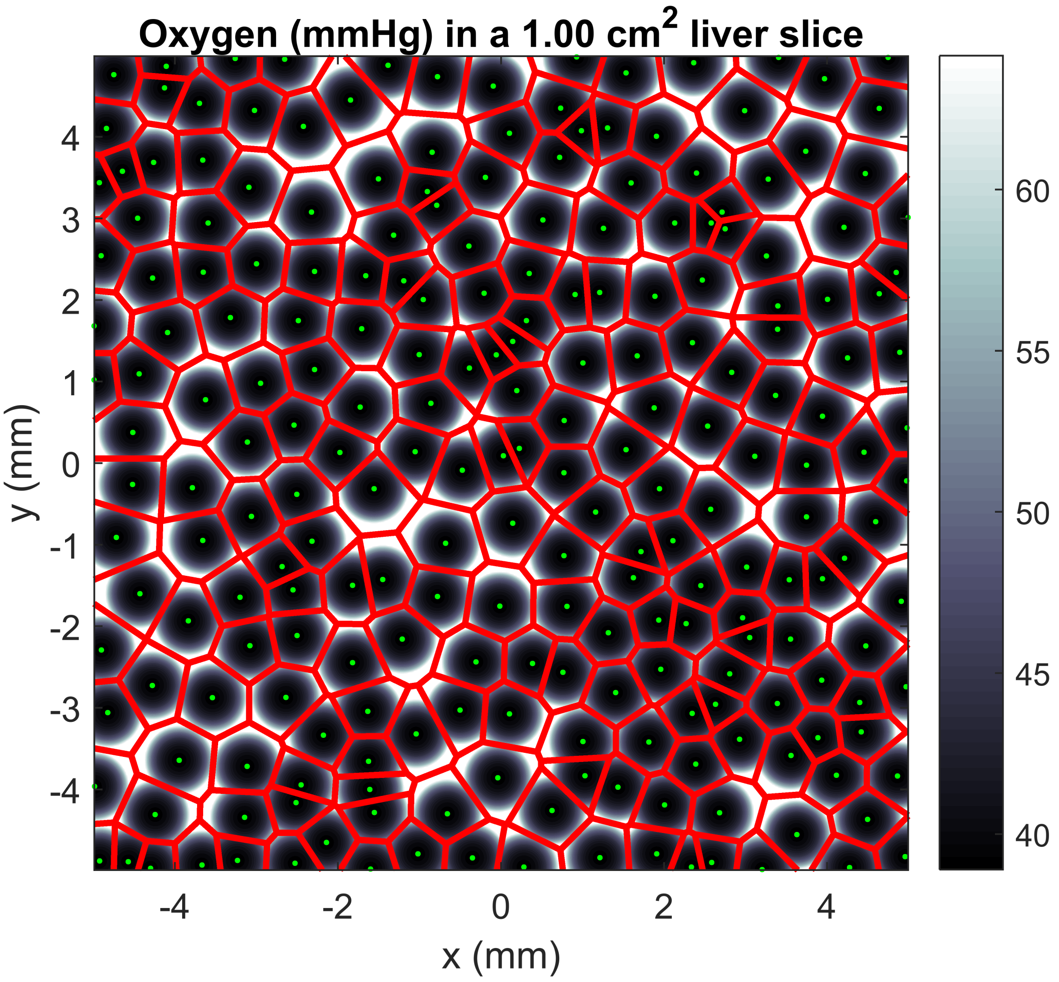

Figure S8. Approximate oxygen distribution (greyscale values) and Voronoi lobule boundaries (red lines) in a 1 cm^2 liver tissue.

Appendix:
Mathematical modeling of liver biology and metastases, and cancer mechanobiology

Liver regeneration

Hoehme et al. used a closed source agent-based modeling (ABM) framework similar to PhysiCell [3] to model liver damage and evaluate the cell-scale hypotheses needed for successful regeneration of the microarchitecture [4]. The model successfully replicated all hepatocytes and sinusoids within a liver lobule during regeneration [4]. More recently, they used a deformable cell-based model to explore how liver lobule cells regenerate with mechanical stress effects [5], finding that the migration forces that a cell needs to exert on its environment are much smaller than those predicted by center-based models that do not explicitly model cell morphologies. (Center-based models are off-lattice methods that track each cell's center of mass and size, typically using one agent to represent one cell [6]).

Liver toxicology

Schwen et al. described a methodology of exploring whole-body pharmacokinetics models based on a liver model, integrating from cells through sinusoids and the organ to the whole organism [7]. Sluka et al. developed a multi-scale, liver-centric physiologic-based pharmacokinetics (PBPK) model to study acetaminophen pharmacology and metabolism [8]. They explored drug uptake and transport at the whole body level, blood flow at the tissue/organ level, and metabolism at the sub-cellular level, showing the model is capable of predicting liver toxicity from acetaminophen overdose.

Interstitial flow and mechanics in liver tissues

Rani et al. presented a computational fluid dynamics (CFD) model of non-Newtonian blood flow within a portal vein, hepatic artery and two sinusoids [9]. Ricken et al. developed a two-scale, continuum multicomponent model for studying blood perfusion and cell metabolism within the liver [10]. They used porous media theory to explore perfusion within lobules, with a focus on advective and diffusive solute transport. Lettmann et al. used a porous-medium method to simulate the microcirculation fields of liver lobules [11]. They captured the blood flow velocity inside the sinusoids and oxygen consumption of Rappaport's liver acinus model. The work found that the liver microcirculation plays an important role in establishing inflammatory cytokine levels.

White et al. presented a convection-diffusion-reaction partial differential equation (PDE) model that coupled blood flow, drug distribution, and clearance in a 3-D liver [12]. Siggers et al. presented a mathematical model that considers the sinusoidal and interstitial spaces as porous media to simulate the blood, interstitial flow and lymph production in the liver [13]. Meyer et al. developed a 3D multi-scale model that explored the biliary fluid dynamics in the mouse liver lobule, predicting gradients of bile velocity and pressure within the lobule [14]. They found that osmotic effects and bile canaliculi contractility result in the bile flow. The multi-scale model is capable of predicting the biliary distribution on drug-induced liver injury. Nishii et al. developed sophisticated poro-viscoelastic (PVE) models that extend porous flow models (as described above) by coupling the flow to a moving, viscoelastic tissue [15]. We shall describe this model further below.

Tissue mechanics in tumor growth

Ambrosi et al. developed an elasto-visco-plastic model that used phase field methods to describe the mechanical stress of tumor cell suspensions and aggregates [16, 17]. The model illustrates tumor growth responses to mechanical stress, cellular release of stresses, the formation of tumor capsules, and compression of neighboring tissues. Antonio et al. introduced an agent-based model for studying elasto-plastic mechanical interactions between cell-basement membrane (BM)-extracellular matrix (ECM) [18]. Cell motion was modeled as resulting from a balance between adhesive, elastic, and repulsive forces. Over short times, elastic forces were used to simulate the interaction between BM and ECM; over long times, the BM underwent plastic relaxation in response to prolonged strains. Liedekerke et al. used an agent-based model to study the tumor spheroids' growth dynamics in mechanical stress environment with calibrated parameters from experiments [19]. They discovered that the growth response of cell populations on external mechanical stress is predicable with a same growth progression function.

Liver metastases in connection with ECM remodeling

Mathematical modeling has also been used to study liver fibrosis [20, 21], while others have used PDE methods to model the growth of liver metastases [22–24]. The kinetics of integrin receptor binding to hepatic ECM proteins following liver injury were recently modeled by Hudson et al. [25]. In follow-on work, the growth of liver metastases was evaluated in a tissue-scale model [26], taking into consideration heterogeneous macrophage populations [27]. ECM from both injured and normal liver was modeled, and parameters were set with values from mouse *in vivo* experiments designed to mimic human tissue conditions.

Mechanosensing and cancer cell proliferation

Others have evaluated the impact of stress on tumor growth and invasion. Voutouri et al. used both in *vivo* experiment and continuum modeling to study the effect of tumor-host mechanical interactions on the growth of solid tumors [28]. The results

show that solid stresses can inhibit tumor cell proliferation, induce apoptosis in surrounding tissue, and lead the surrounding tissue to resist tumor expansion. Cheng et al. [29] built an experimental model to explore spatial distribution of compressive stress around tumor spheroids representing avascular nodules, showing that high mechanical stress results in suppression of tumor cell proliferation and apoptotic cell death.

Metastasis

While there has been substantial work in modeling primary tumor growth in detailed tissues [6, 30], to our knowledge there has been little work in spatial models of early metastatic seeding and growth under mechanical and flow constraints, especially in liver tissue. Macklin and Lowengrub used a level set / moving boundary method to model tumor growth in simulated brain tissue in [31], but they did not consider mechanobiologic feedback or the impact of flow. Their continuum approach was also not suited to tumors with low cell-cell adhesion or high cellular heterogeneity. Baratchart et al. recently developed a similar model (also based on a porous flow formulation, but without the level set-based sharp interface model) to simulate the merger of renal metastatic foci [32], with a very simple pressure-based tumor cell proliferation rate, and without considering any mechanobiologic feedback in the surrounding normal tissue.

Others have developed network models of metastatic seeding [33–38], but without spatially-resolved simulation of the individual metastasis sites, nor of any mechanical effects. Treatment of liver metastases via nanotherapy taking into account immune system interactions have been recently modeled [39–41], but without considering mechanical effects. In particular, detection and drug delivery to colorectal cancer liver metastases via nanoparticles were simulated in a tissue-scale model in [42, 43]. See [44] for further excellent materials on experimental and mathematical modeling of metastasis.

Appendix:
Details on dimensional analysis of PVE model

To evaluate the relative contribution of the advective and diffusive effects in liver interstitial fluid flow, we can rewrite Equations 1-2 as

$$\frac{\partial \sigma}{\partial t} = D\nabla^2\sigma - \vec{u}\cdot\nabla\sigma - \sigma\nabla\cdot\vec{u} - \lambda\sigma. \tag{8}$$

Approximating the flow field as slowly varying ($\vec{u} \approx u\vec{w}$ for some constant u and a constant unit vector $\vec{w}$), $\nabla\cdot\vec{u} \approx 0$. If we nondimensionalize space with scale L and time with scale $\bar{t}$, then this becomes

$$\frac{\partial \sigma}{\partial t} = \left(\frac{D\bar{t}}{L^2}\right)\nabla^2\sigma - \left(\frac{u\bar{t}}{L}\right)\vec{w}\cdot\nabla\sigma - (\lambda\bar{t})\,\sigma. \tag{9}$$

Choosing

$$\frac{u\bar{t}}{L} = 1 \text{ and } \lambda\bar{t} = 1 \Longrightarrow \bar{t} = \frac{1}{\lambda} \text{ and } L = \frac{u}{\lambda}, \tag{10}$$

then the nondimensionalized equation becomes

$$\frac{\partial \sigma}{\partial t} = \left(\frac{D\lambda}{u^2}\right)\nabla^2\sigma - \vec{w}\cdot\nabla\sigma - \sigma. \tag{11}$$

By prior work [45], $D \sim 10^5\mu\text{m}^2/\text{min}$ and $\lambda \sim 10\ \text{min}^{-1}$. By our PVE simulations of a control case (with no tumor cells) and with small micrometastases, as well as the simulations in [15], $u \sim 10^{-4}\text{m/sec} = 6\times10^3\mu\text{m/min}$ in the unobstructed liver parenchyma, and so $L \sim 600\,\mu\text{m}$, and $\bar{t} \sim 6$ sec. The diffusion term has relative order of magnitude

$$\frac{D\lambda}{u^2} \sim \frac{1}{36} \approx 0.03. \tag{12}$$

Thus, in unobstructed regions of liver lobule, flow is primarily advective-reactive, and the original equation can be simplified to

$$\frac{\partial \sigma}{\partial t} = -\nabla\cdot(\sigma\vec{u}) - \lambda\sigma. \tag{13}$$

Because the time scale is on the order of seconds, advective flow is at quasi-steady state in regions of unobstructed flow when considered on time scales of mechanics (minutes) and tumor growth (hours to days).

Because $\vec{u} \sim \mathbf{0}$ inside micrometastases, we find that biotransport in those regions satisfies

$$\frac{\partial \sigma}{\partial t} \approx D\nabla^2\sigma - \lambda\sigma. \tag{14}$$

That is, biotransport is largely diffusive-reactive within tumors.

Appendix:
Details on radial biotransport approximation

Here, we give expanded mathematical detail on the approximate analytical solutions for oxygen distribution in unobstructed liver tissue.

Assuming incompressible flow ($\nabla \cdot \vec{u} = 0$) and that flow within the lobule can be approximated as radially symmetric ($\vec{u} \approx -u(r)\hat{\vec{r}}$, where r is the distance from the middle of the central vein), we have

$$\frac{\partial \sigma}{\partial t} = u(r)\nabla\sigma \cdot \hat{\vec{r}} - \lambda\sigma \quad (15)$$

$$= u(r)\frac{\partial \sigma}{\partial r} - \lambda\sigma. \quad (16)$$

Under quasi-steady conditions, $\sigma = \sigma(r)$, and we have

$$0 = \sigma'(r) - \frac{\lambda}{u(r)}\sigma, \quad (17)$$

whose analytical solution is given by

$$\sigma(r) = ce^{\lambda \int_0^r \frac{1}{u(s)} ds} \quad (18)$$

for some constant c.

For simplicity, we will approximate the flow profiles shown for no tumor cells or with small micrometastases with the form

$$u(r) \approx ae^{-br} \quad (19)$$

for constants a and b. Notice that $u(0) = a$ and $u(R) = ae^{-bR} = u(0)e^{-bR}$, whereby

$$b = -\frac{\ln\left(\frac{u(R)}{u(0)}\right)}{R}. \quad (20)$$

For consistency with the PVE model, we set $R = 400\,\mu$m.

For this simplified form, we have

$$\sigma(r) \approx ce^{\frac{\lambda}{ab}\left(e^{br}-1\right)}. \quad (21)$$

By our PVE simulations for the control with no tumor cells or with small micrometastases, we see that $\frac{u(R)}{u(0)}$ is on the order of 0.01 to 0.1, so we choose 0.05. Thus, $b \sim 0.0075\,\mu\mathrm{m}^{-1}$ by Equation 20. Moreover, $u(0) = a \sim 10^{-3}\,\mathrm{m/sec} = 6 \times 10^4\,\mu\mathrm{m/min}$. [2] report that liver oxygenation is approximately 38.9 mmHg in central venules, 48.2 mmHg in sinusoids, and 59.8 mmHg in portal venules. So, we set $\sigma(0) = 38.9$ mmHg:

Appendix:
Code availability

.1 Poroviscoelastic model

The project files for the poroviscoelastic simulation work are available as open source under the MIT license at

https://github.com/MathCancer/PVE-liver-mets

Zenodo archives

1.0.0 Version used for the PVE simulation studies
https://doi.org/10.5281/zenodo.3779512

Agent-based model

The core agent-based model (minus the cloud-hosted interface) is available as open source under the 3-clause BSD license at

https://github.com/MathCancer/liver-mechanobiology

Zenodo archives

1.0.0 Version used for the main parameter studies
https://doi.org/10.5281/zenodo.3766870

1.1.0 Update to allow resizeable domains
https://doi.org/10.5281/zenodo.3766979

nanoHUB cloud-hosted model

The cloud-hosted deployment of the agent-based model can be accessed and run for free at

https://nanohub.org/tools/pc4livermedium.

The source code is available as open source under the 3-clause BSD license at

https://github.com/yafeiwang89/liver_medium

Zenodo archives

1.3 Source code for nanoHUB app version 1.3
http://doi.org/10.5281/zenodo.3775924

References

1. Macklin, P. & Lowengrub, J. S. Nonlinear simulation of the effect of microenvironment on tumor growth. *J. Theor. Biol.* **245**, 677–704, DOI: 10.1016/j.jtbi.2006.12.004 (2007).
2. Tsukada, K. & Suematsu, M. Visualization and analysis of blood flow and oxygen consumption in hepatic microcirculation: Application to an acute hepatitis model. *J. Vis. Exp.* **66**, e3996, DOI: 10.3791/3996 (2012).
3. Ghaffarizadeh, A., Heiland, R., Friedman, S. H., Mumenthaler, S. M. & Macklin, P. PhysiCell: an open source physics-based cell simulator for 3-D multicellular systems. *PLoS Comput. Biol.* **14**, e1005991, DOI: 10.1371/journal.pcbi.1005991 (2018).
4. Hoehme, S. *et al.* Prediction and validation of cell alignment along microvessels as order principle to restore tissue architecture in liver regeneration. *Proc. Natl. Acad. Sci.* **107**, 10371–10376, DOI: 10.1073/pnas.0909374107 (2010).
5. Van Liedekerke, P. *et al.* Quantifying the mechanics and growth of cells and tissues in 3d using high resolution computational models. *bioRxiv* 470559, DOI: 10.1101/470559 (2018).
6. Metzcar, J., Wang, Y., Heiland, R. & Macklin, P. A review of cell-based computational modeling in cancer biology. *JCO Clin. Cancer Informatics* **3**, 1–13, DOI: 10.1200/CCI.18.00069 (2019). (invited review).
7. Schwen, L. O. *et al.* Representative sinusoids for hepatic four-scale pharmacokinetics simulations. *PloS one* **10**, e0133653, DOI: 10.1371/journal.pone.0133653 (2015).
8. Sluka, J. P. *et al.* A liver-centric multiscale modeling framework for xenobiotics. *PloS one* **11**, e0162428, DOI: 10.1371/journal.pone.0162428 (2016).
9. Rani, H., Sheu, T. W., Chang, T. & Liang, P. Numerical investigation of non-newtonian microcirculatory blood flow in hepatic lobule. *J. biomechanics* **39**, 551–563, DOI: 10.1016/j.jbiomech.2004.11.029 (2006).
10. Ricken, T. *et al.* Modeling function–perfusion behavior in liver lobules including tissue, blood, glucose, lactate and glycogen by use of a coupled two-scale pde–ode approach. *Biomech. modeling mechanobiology* **14**, 515–536, DOI: 10.1007/s10237-014-0619-z (2015).
11. Lettmann, K. A. & Hardtke-Wolenski, M. The importance of liver microcirculation in promoting autoimmune hepatitis via maintaining an inflammatory cytokine milieu–a mathematical model study. *J. theoretical biology* **348**, 33–46, DOI: 10.1016/j.jtbi.2014.01.016 (2014).
12. White, D., Coombe, D., Rezania, V. & Tuszynski, J. Building a 3d virtual liver: Methods for simulating blood flow and hepatic clearance on 3d structures. *PloS one* **11**, e0162215, DOI: 10.1371/journal.pone.0162215 (2016).
13. Siggers, J. H., Leungchavaphongse, K., Ho, C. H. & Repetto, R. Mathematical model of blood and interstitial flow and lymph production in the liver. *Biomech. modeling mechanobiology* **13**, 363–378, DOI: 10.1007/s10237-013-0516-x (2014).
14. Meyer, K. *et al.* A predictive 3d multi-scale model of biliary fluid dynamics in the liver lobule. *Cell systems* **4**, 277–290, DOI: 10.1016/j.cels.2017.02.008 (2017).
15. Nishii, K., Reese, G., Moran, E. C. & Sparks, J. L. Multiscale computational model of fluid flow and matrix deformation in decellularized liver. *J. mechanical behavior biomedical materials* **57**, 201–214, DOI: 10.1016/j.jmbbm.2015.11.033 (2016).
16. Ambrosi, D. & Preziosi, L. Cell adhesion mechanisms and stress relaxation in the mechanics of tumours. *Biomech. modeling mechanobiology* **8**, 397, DOI: 10.1007/s10237-008-0145-y (2009).
17. Preziosi, L., Ambrosi, D. & Verdier, C. An elasto-visco-plastic model of cell aggregates. *J. theoretical biology* **262**, 35–47, DOI: 10.1016/j.jtbi.2009.08.023 (2010).
18. D'Antonio, G., Macklin, P. & Preziosi, L. An agent-based model for elasto-plastic mechanical interactions between cells, basement membrane and extracellular matrix. *Math. Biosci. Eng.* **10**, 75–101, DOI: 10.3934/mbe.2013.10.75 (2013).
19. Van Liedekerke, P. *et al.* Quantitative cell-based model predicts mechanical stress response of growing tumor spheroids over various growth conditions and cell lines. *PLoS computational biology* **15**, e1006273, DOI: 10.1371/journal.pcbi.1006273 (2019).

20. Friedman, A. & Hao, W. Mathematical modeling of liver fibrosis. *Math. Biosci. & Eng.* **14**, 143–164, DOI: 10.3934/mbe.2017010 (2017).

21. Dutta-Moscato, J. *et al.* A multiscale agent-based in silico model of liver fibrosis progression. *Front. bioengineering biotechnology* **2**, 18, DOI: 10.3389/fbioe.2014.00018 (2014).

22. Haeno, H. *et al.* Computational modeling of pancreatic cancer reveals kinetics of metastasis suggesting optimum treatment strategies. *Cell* **148**, 362–375, DOI: 10.1016/j.cell.2011.11.060 (2012).

23. Campbell, A., Sivakumaran, T., Davidson, M., Lock, M. & Wong, E. Mathematical modeling of liver metastases tumour growth and control with radiotherapy. *Phys. Medicine & Biol.* **53**, 7225, DOI: 10.1088/0031-9155/53/24/015 (2008).

24. Bethge, A., Schumacher, U. & Wedemann, G. Simulation of metastatic progression using a computer model including chemotherapy and radiation therapy. *J. biomedical informatics* **57**, 74–87, DOI: 10.1016/j.jbi.2015.07.011 (2015).

25. Hudson, S. V. *et al.* Modeling the kinetics of integrin receptor binding to hepatic extracellular matrix proteins. *Sci. reports* **7**, 12444, DOI: 10.1038/s41598-017-12691-y (2017).

26. Hudson, S. V. *et al.* Computational/experimental evaluation of liver metastasis post hepatic injury: interactions with macrophages and transitional ecm. *Sci. Reports* **9**, 1–13, DOI: 10.1038/s41598-019-51249-y (2019).

27. Mahlbacher, G., Curtis, L. T., Lowengrub, J. & Frieboes, H. B. Mathematical modeling of tumor-associated macrophage interactions with the cancer microenvironment. *J. for immunotherapy cancer* **6**, 10, DOI: 10.1186/s40425-017-0313-7 (2018).

28. Voutouri, C., Mpekris, F., Papageorgis, P., Odysseos, A. D. & Stylianopoulos, T. Role of constitutive behavior and tumor-host mechanical interactions in the state of stress and growth of solid tumors. *PloS one* **9**, e104717, DOI: 10.1371/journal.pone.0104717 (2014).

29. Cheng, G., Tse, J., Jain, R. K. & Munn, L. L. Micro-environmental mechanical stress controls tumor spheroid size and morphology by suppressing proliferation and inducing apoptosis in cancer cells. *PLoS one* **4**, e4632, DOI: 10.1371/journal.pone.0004632 (2009).

30. Chamseddine, I. M. & Rejniak, K. A. Hybrid modeling frameworks of tumor development and treatment. *WIREs Syst. Biol. Medicine* **12**, e1461, DOI: 10.1002/wsbm.1461 (2020). https://onlinelibrary.wiley.com/doi/pdf/10.1002/wsbm.1461.

31. Macklin, P. & Lowengrub, J. S. A New Ghost Cell/Level Set Method for Moving Boundary Problems: Application to Tumor Growth. *J. Sci. Comput.* **35**, 266–299, DOI: 10.1007/s10915-008-9190-z (2008). (invited author: J.S. Lowengrub).

32. Baratchart, E. *et al.* Computational modelling of metastasis development in renal cell carcinoma. *PLoS computational biology* **11**, e1004626, DOI: 10.1371/journal.pcbi.1004626 (2015).

33. Scott, J. G., Basanta, D., Anderson, A. R. & Gerlee, P. A mathematical model of tumour self-seeding reveals secondary metastatic deposits as drivers of primary tumour growth. *J. The Royal Soc. Interface* **10**, 20130011, DOI: 10.1098/rsif.2013.0011 (2013).

34. Haeno, H. & Michor, F. The evolution of tumor metastases during clonal expansion. *J. theoretical biology* **263**, 30–44, DOI: 10.1016/j.jtbi.2009.11.005 (2010).

35. Newton, P. K. *et al.* Spreaders and sponges define metastasis in lung cancer: a markov chain monte carlo mathematical model. *Cancer research* **73**, 2760–2769, DOI: 10.1158/0008-5472.CAN-12-4488 (2013).

36. Scott, J. G., Fletcher, A. G., Maini, P. K., Anderson, A. R. & Gerlee, P. A filter-flow perspective of haematogenous metastasis offers a non-genetic paradigm for personalised cancer therapy. *Eur. journal cancer* **50**, 3068–3075, DOI: 10.1016/j.ejca.2014.08.019 (2014).

37. Newton, P. K. *et al.* Spatiotemporal progression of metastatic breast cancer: a markov chain model highlighting the role of early metastatic sites. *NPJ breast cancer* **1**, 15018, DOI: 10.1038/npjbcancer.2015.18 (2015).

38. Newton, P. K. *et al.* A stochastic markov chain model to describe lung cancer growth and metastasis. *PloS one* **7**, e34637, DOI: 10.1371/journal.pone.0034637 (2012).

39. Leonard, F. *et al.* Enhanced performance of macrophage-encapsulated nanoparticle albumin-bound-paclitaxel in hypo-perfused cancer lesions. *Nanoscale* **8**, 12544–12552, DOI: 10.1039/C5NR07796F (2016).

40. Leonard, F. *et al.* Macrophage polarization contributes to the anti-tumoral efficacy of mesoporous nanovectors loaded with albumin-bound paclitaxel. *Front. immunology* **8**, 693, DOI: 10.3389/fimmu.2017.00693 (2017).

41. Leonard, F. *et al.* Nonlinear response to cancer nanotherapy due to macrophage interactions revealed by mathematical modeling and evaluated in a murine model via crispr-modulated macrophage polarization. *Cancer Immunol. Immunother.* **69**, 731–744, DOI: 10.1007/s00262-020-02504-z (2020).

42. Curtis, L. T., Rychahou, P., Bae, Y. & Frieboes, H. B. A computational/experimental assessment of antitumor activity of polymer nanoassemblies for ph-controlled drug delivery to primary and metastatic tumors. *Pharm. Res.* **33**, 2552–2564, DOI: 10.1007/s11095-016-1981-6 (2016).

43. Reichel, D. *et al.* Development of halofluorochromic polymer nanoassemblies for the potential detection of liver metastatic colorectal cancer tumors using experimental and computational approaches. *Pharm. Res.* **34**, 2385–2402, DOI: 10.1007/s11095-017-2245-9 (2017).

44. Malek, A. *Experimental metastasis: modeling and analysis* (Springer, 2013).

45. Ghaffarizadeh, A., Friedman, S. H. & Macklin, P. BioFVM: an efficient, parallelized diffusive transport solver for 3-D biological simulations. *Bioinformatics* **32**, 1256–8, DOI: 10.1093/bioinformatics/btv730 (2016).
