# Supplementary material for: Impact of tumor-parenchyma biomechanics on liver metastatic progression: a multi-model approach
Source: Sci Rep. 2021 Jan 18;11:1710. doi: 10.1038/s41598-020-78780-7 (PMC7813881; doi:10.1038/s41598-020-78780-7)
Supplement: Supplementary file 2 — Supplementary Information 2. [file 41598_2020_78780_MOESM2_ESM.pdf]

Supplementary Materials for PVE MODEL:

Impact of tumor-parenchyma biomechanics on liver metastatic progression: a multi-model approach

Yafei Wang[†], Erik Brodin[†], Kenichiro Nishii, Hermann B Frieboes, Shannon Mumenthaler, Jessica L. Sparks[1], Paul Macklin[2]

[†]Contributed equally to this work
[1]Corresponding author for poroviscoelastic model. E-mail: sparksj6@MiamiOH.edu
[2] Corresponding author for agent-based model and overall study. E-mail: macklinp@iu.edu

1. Material Properties

Model material properties are given in Tables S1-S3. The Young's modulus of the parenchyme was taken as 4.4 kPa and Poisson's ratio as 0.35 (Evans et al. 2013; Nishii et al. 2016). The Young's modulus for tumor tissue was set as 30 kPa (Venkatesh et al. 2008; Lu et al. 2015) and Poisson's ratio was assumed equal to normal tissue. A four-term Prony series expansion was used to model tissue viscoelasticity, with Prony series constants taken from previous nano-indentation experiments on perfused liver tissue (Evans et al. 2013).

At the macroscopic (cm) length scale, tumors have been reported to have higher hydraulic conductivity *K* relative to the surrounding tissue (Swabb et al. 1974; Netti et al. 2000; Pishko et al. 2011). However, the hydraulic conductivity of smaller tumors on the order of 50-400 μm is unknown. For each tumor size/location combination in the present study, we simulated the effects of high and low hydraulic conductivity in the tumor. For the high hydraulic conductivity case, we used a value of 3.65 times that of normal liver, based on hydraulic conductivity of hepatocarcinoma determined from measurements of (Swabb et al. 1974). For the low hydraulic conductivity case, we used 0.3 times that of normal liver, to span approximately one order of magnitude in our high/low conditions. Normal liver parenchyme hydraulic conductivity was taken as 1.85×10^{-6} m/s as reported by (Nishii et al. 2016), based on void ratio measurements from histological image analysis. Void ratio, e, is defined as:

$$e = \frac{V_v}{V_s} = \frac{V_v}{V_t - V_v} \quad \text{(Eq. S1)}$$

where e is the void ratio, V_v is the void volume, V_s is the volume of solid in the lobule, and V_t is the total volume of the lobule. Void ratio is related to hydraulic conductivity as follows (Nishii et al. 2016):

$$K = \frac{\rho g e r^2}{8\mu(1+e)} \quad \text{(Eq. S2)}$$

where *K* is the hydraulic conductivity (m/s), ρ is fluid density (kg/m^3), g is gravitational acceleration (m/s^2), e is the void ratio, r is the average radius of a sinusoid (m), and μ is the dynamic viscosity of the permeating fluid (Pa·s).

Table S1. Elastic constants for parenchyme and tumor. Materials are assumed isotropic.

	Parenchyme	Tumor
Elastic Modulus (kPa)	4.4	30
Poisson's ratio	0.35	0.35

Table S2. Viscoelastic constants (same values used for parenchyme and tumor).

Term	g_i (dim)	τ_i (sec)
1	0.53	2.00E-05
2	0.376	1
3	0.027	7.65
4	0.01	100

Table S3. Hydraulic conductivity and related properties

Property	Parenchyme	Tumor, High Hydraulic Conductivity	Tumor, Low Hydraulic Conductivity
Hydraulic Conductivity (m/s)	1.85E-06	6.75E-06	5.50E-07
Void Ratio	0.8	1.55	0.052
Specific Weight of Fluid (kg/m^3)	9855	9855	9855

2. Boundary Conditions

As noted in the manuscript, one-quarter of the hexagonal lobule was modeled to minimize computational cost; therefore symmetric boundary conditions were applied to the *X*, *Y*, and *Z* planes (see Model Geometry). Following the work of (Bonfiglio et al. 2010), the model assumed uniform expansion in the axial direction (in the *XY* plane) and no expansion in the longitudinal (*Z*) direction. Pressure in the CV surface was set to zero to serve as a pressure sink, and pressures in the terminal portal vein (tPV) and pre-terminal portal vein (pre-tPV) were set to 2.23 mmHg (297.3 Pa) above the CV pressure so that the physiological pressure difference was preserved (Nishii et al. 2016). The pressure condition at the tumor boundary varied depending on tumor size (Table S4). A total of 13 model runs were performed, varying tumor position and size, pressure condition at the tumor boundary, and tumor permeability relative to surrounding parenchyme (Table S4). Lastly, a control model was created with no tumor but with identical parenchyme properties and vascular pressure boundary conditions, to serve as a basis for comparison.

Table S4. Variable parameters for finite element model runs.

Model Run	Tumor Position	Tumor Size (diam., μm)	Tumor as Pressure Source or Sink (Pa)	Tumor Hydraulic Conductivity (m/s)
1	Center	Seed (50)	Neutral	Low (5.50E-07)
2	Center	Seed (50)	Neutral	High (6.75E-06)
3	Center	Small (200)	Sink (0)	Low (5.50E-07)
4	Center	Small (200)	Sink (0)	High (6.75E-06)
5	Portal	Seed (50)	Neutral	Low (5.50E-07)
6	Portal	Seed (50)	Neutral	High (6.75E-06)
7	Portal	Small (200)	Sink (0)	Low (5.50E-07)
8	Portal	Small (200)	Sink (0)	High (6.75E-06)
9	Portal	Medium (400)	Source (600)	Low (5.50E-07)
10	Portal	Medium (400)	Source (600)	High (6.75E-06)
11	Portal	Medium (400)	Neutral	Low (5.50E-07)
12	Portal	Medium (400)	Neutral	High (6.75E-06)
13	Control	No Tumor	N/A	Parenchyme (1.85E-06)

3. Fluid Velocity, Strain, Stress, and Pore Fluid Pressure Results

(A)

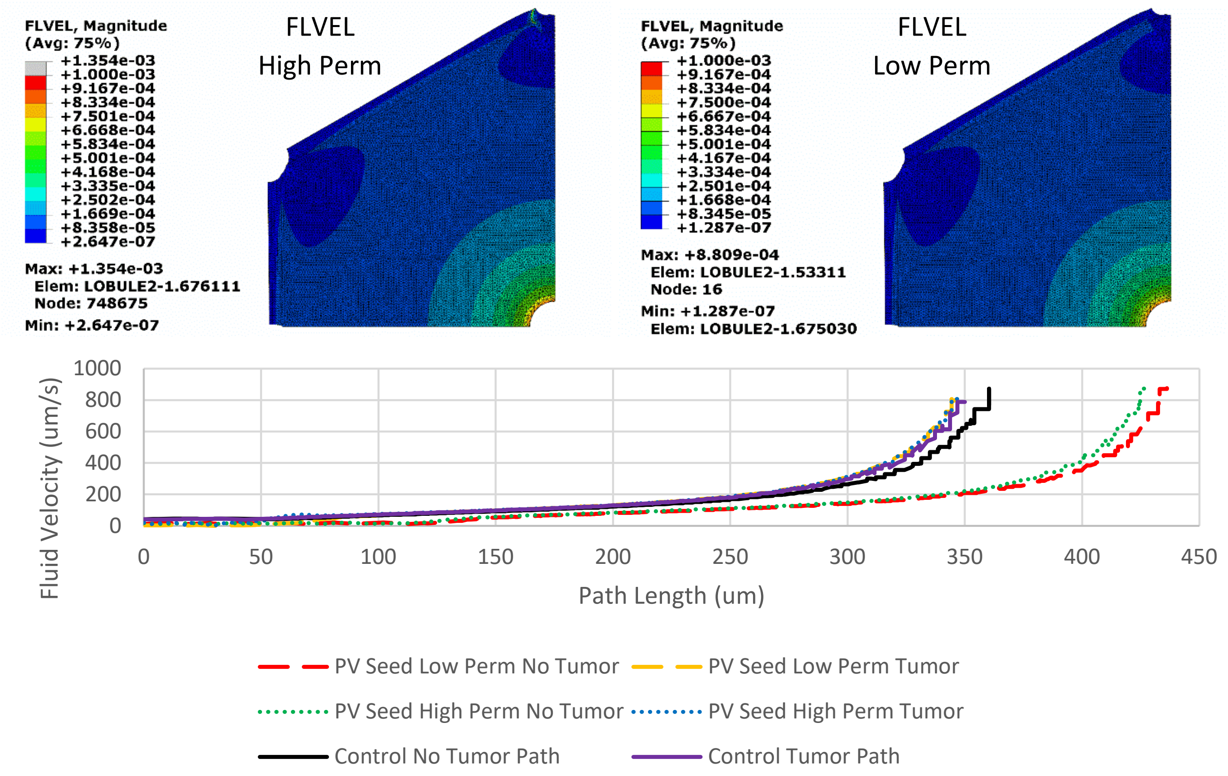

(B)

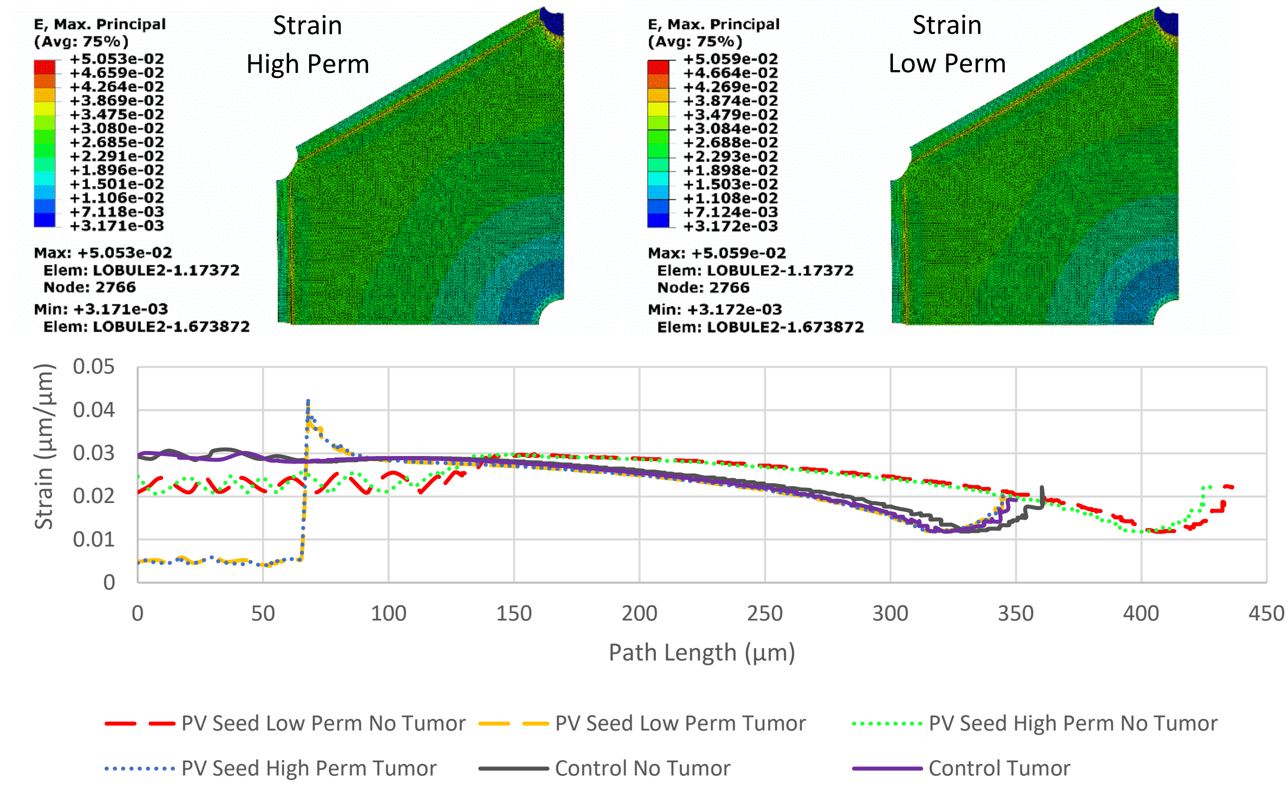

(C)

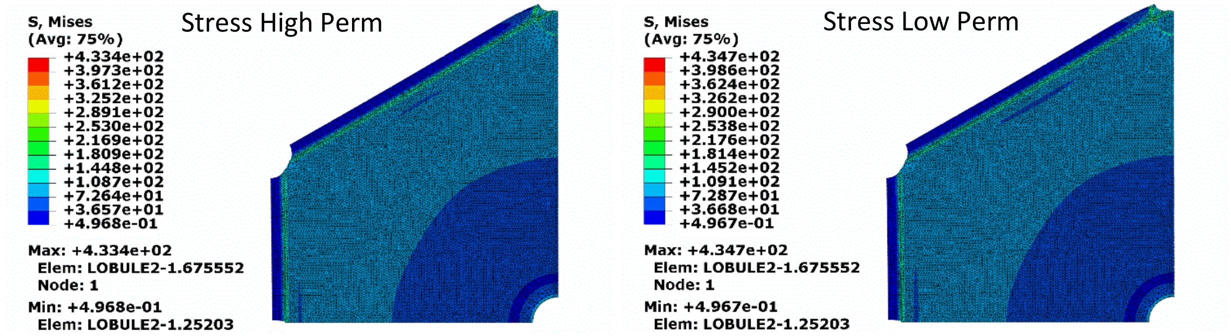

(D)

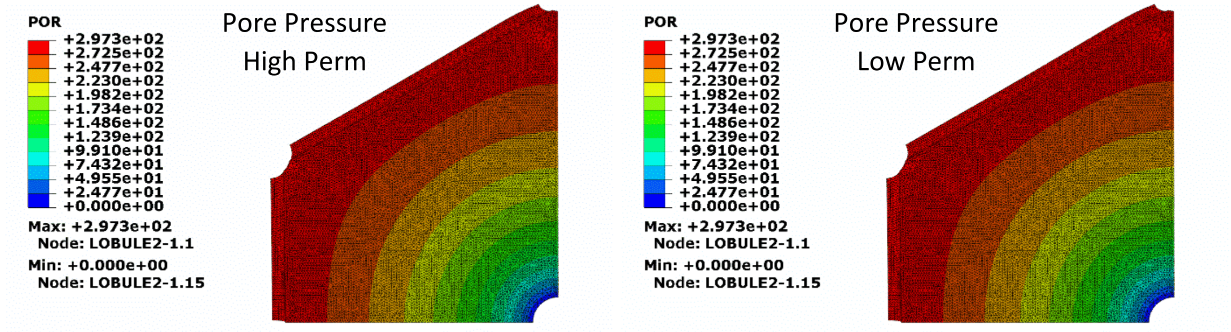

Figure S9. Images for the 50μm diameter tumor seed centered at the portal vein. Tumor was not set as a pressure source or sink. Images for (A) fluid velocity, (B) strain, (C) stress, and (D) pore pressure distribution throughout the quarter lobule. Graphs represent the fluid velocity and strain in the “tumor” and “no tumor” paths.

(A)

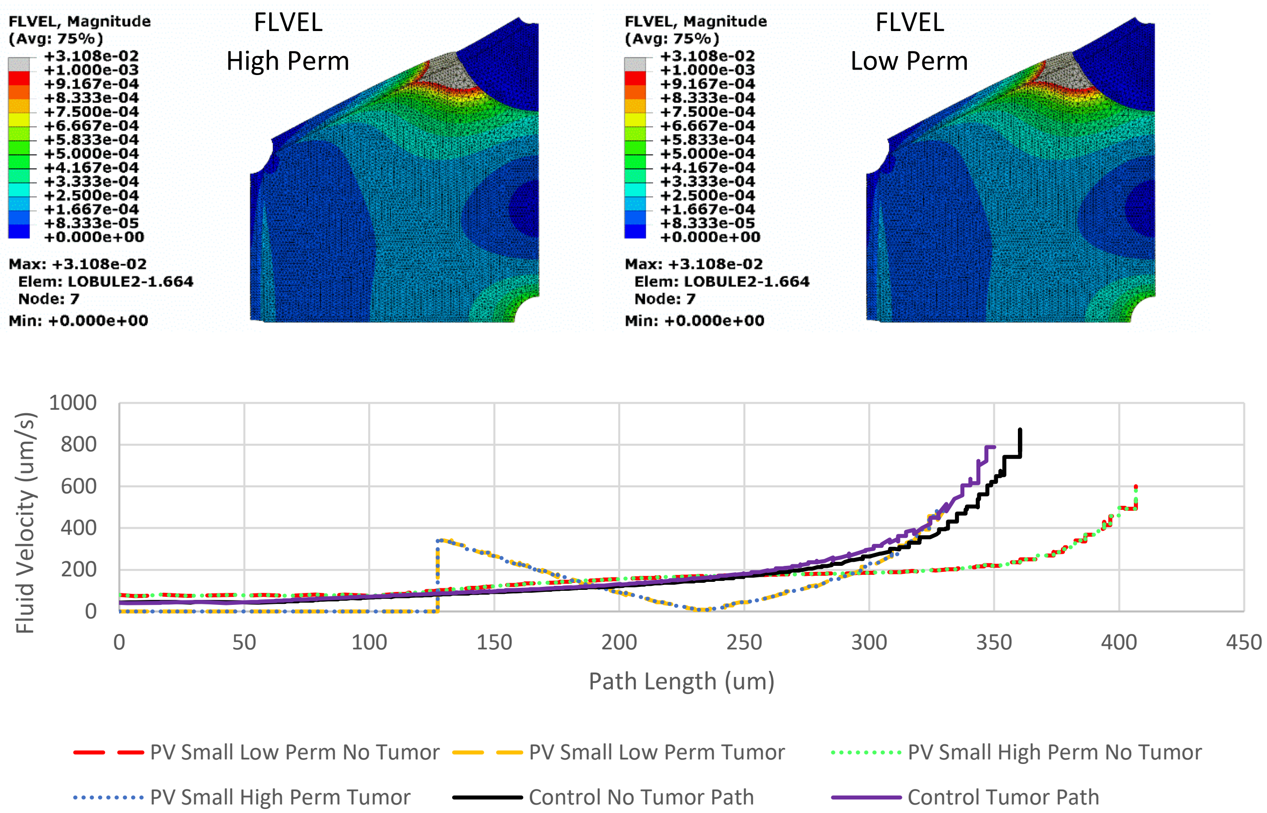
FLVEL, Magnitude
(Avg: 75%)
+3.108e-02
+1.000e-03
+9.167e-04
+8.333e-04
+7.500e-04
+6.667e-04
+5.833e-04
+5.000e-04
+4.167e-04
+3.333e-04
+2.500e-04
+1.667e-04
+8.333e-05
+0.000e+00
Max: +3.108e-02
Elem: LOBULE2-1.664
Node: 7
Min: +0.000e+00
FLVEL
High Perm
FLVEL, Magnitude
(Avg: 75%)
+3.108e-02
+1.000e-03
+9.167e-04
+8.333e-04
+7.500e-04
+6.667e-04
+5.833e-04
+5.000e-04
+4.167e-04
+3.333e-04
+2.500e-04
+1.667e-04
+8.333e-05
+0.000e+00
Max: +3.108e-02
Elem: LOBULE2-1.664
Node: 7
Min: +0.000e+00
FLVEL
Low Perm
Fluid Velocity (um/s)
1000
800
600
400
200
0
0
50
100
150
200
250
300
350
400
450
Path Length (um)
PV Small Low Perm No Tumor
PV Small Low Perm Tumor
PV Small High Perm No Tumor
PV Small High Perm Tumor
Control No Tumor Path
Control Tumor Path

(B)

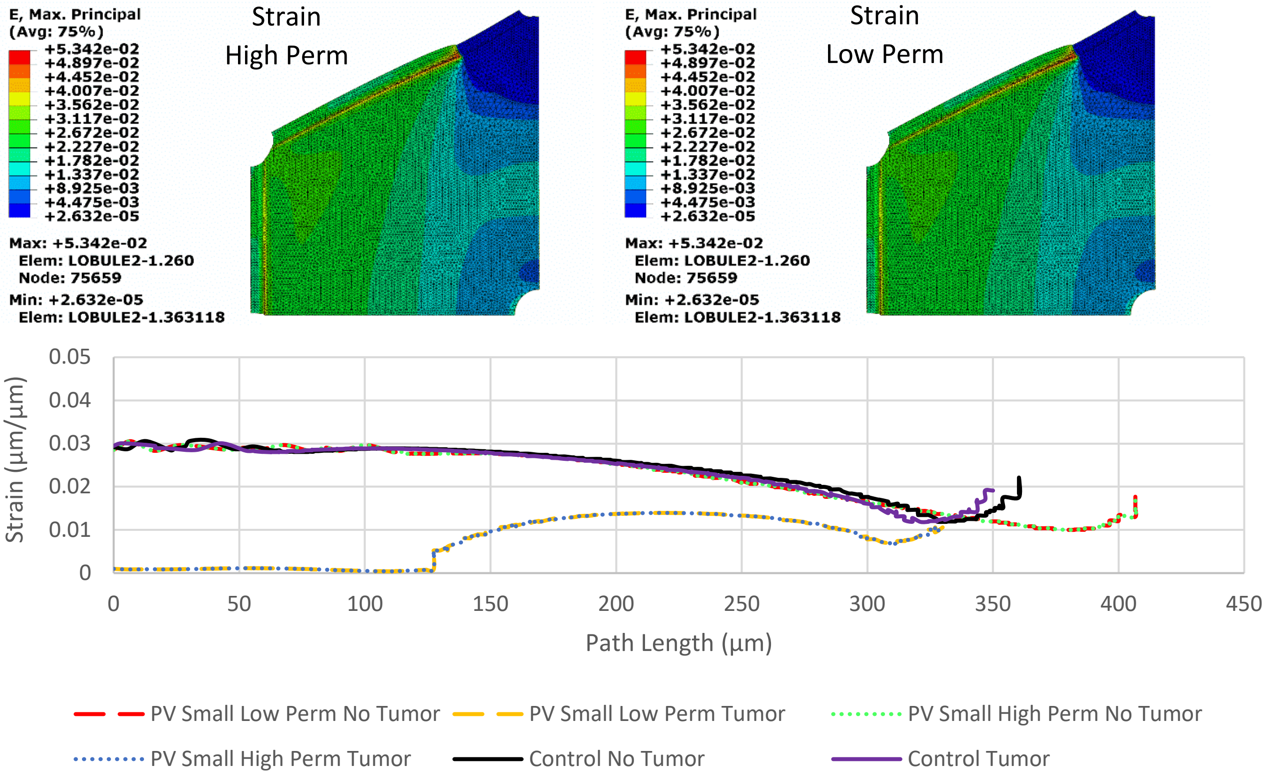
E, Max. Principal
(Avg: 75%)
+5.342e-02
+4.897e-02
+4.452e-02
+4.007e-02
+3.562e-02
+3.117e-02
+2.672e-02
+2.227e-02
+1.782e-02
+1.337e-02
+8.925e-03
+4.475e-03
+2.632e-05
Max: +5.342e-02
Elem: LOBULE2-1.260
Node: 75659
Min: +2.632e-05
Elem: LOBULE2-1.363118
Strain
High Perm
E, Max. Principal
(Avg: 75%)
+5.342e-02
+4.897e-02
+4.452e-02
+4.007e-02
+3.562e-02
+3.117e-02
+2.672e-02
+2.227e-02
+1.782e-02
+1.337e-02
+8.925e-03
+4.475e-03
+2.632e-05
Max: +5.342e-02
Elem: LOBULE2-1.260
Node: 75659
Min: +2.632e-05
Elem: LOBULE2-1.363118
Strain
Low Perm
Strain (µm/µm)
0.05
0.04
0.03
0.02
0.01
0
0
50
100
150
200
250
300
350
400
450
Path Length (µm)
PV Small Low Perm No Tumor
PV Small Low Perm Tumor
PV Small High Perm No Tumor
PV Small High Perm Tumor
Control No Tumor
Control Tumor

(C)

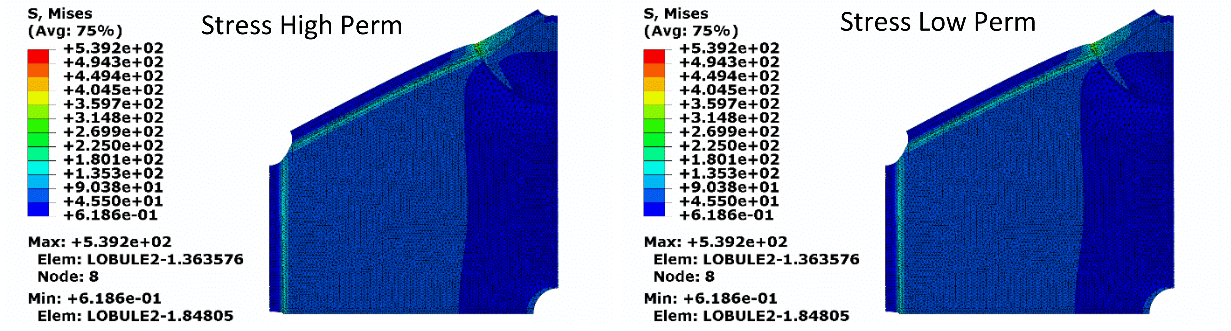

(D)

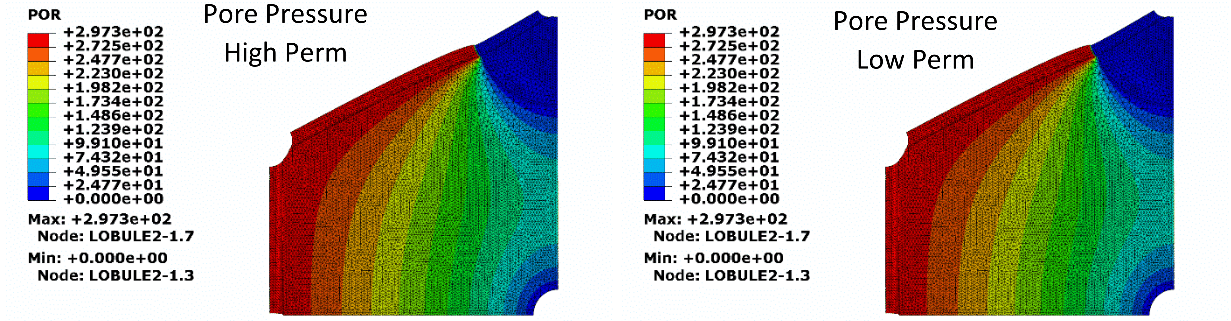

Figure S10. Images for the 200μm diameter tumor centered at the portal vein. Tumor was set as a pressure sink. Images for (A) fluid velocity, (B) strain, (B) stress, and (D) pore pressure distribution throughout the quarter lobule. Graphs represent the fluid velocity and strain in the “tumor” and “no tumor” paths.

(A)

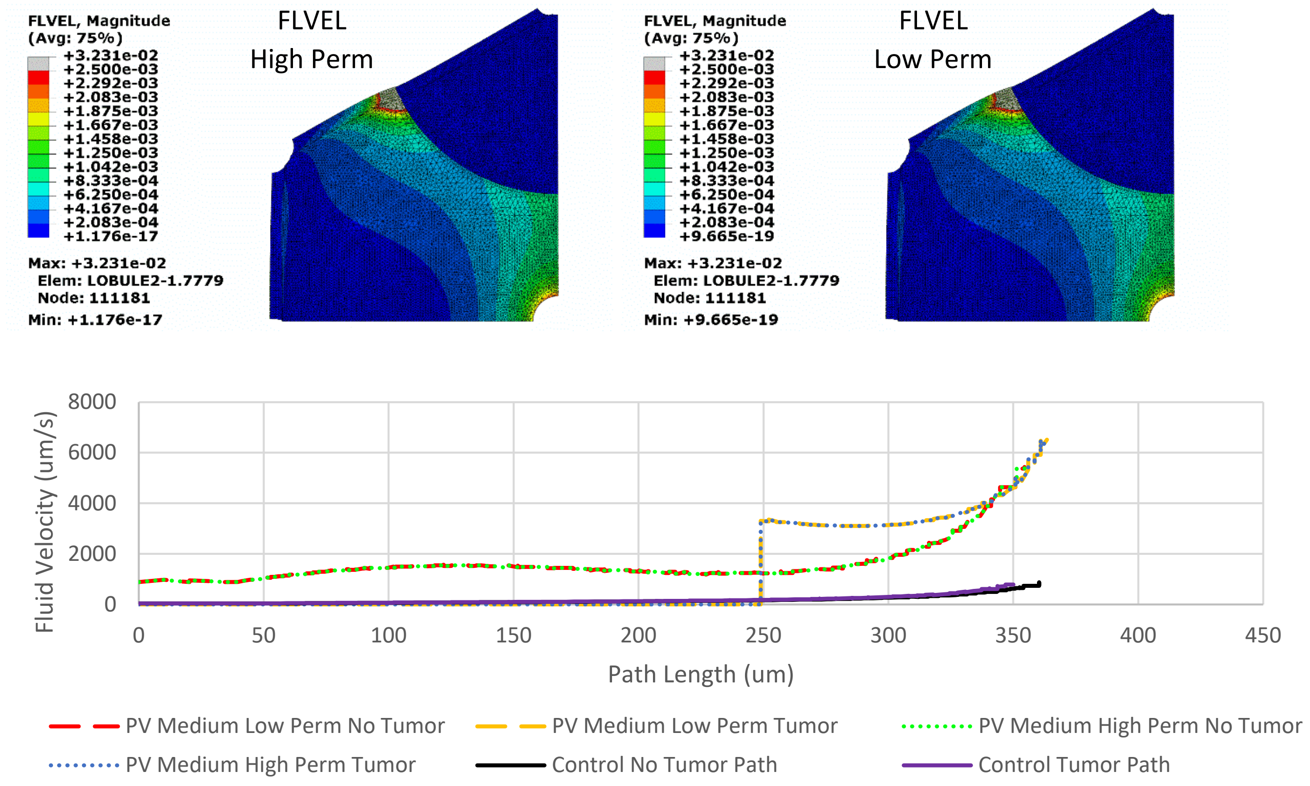
FLVEL, Magnitude
(Avg: 75%)
+3.231e-02
+2.500e-03
+2.292e-03
+2.083e-03
+1.875e-03
+1.667e-03
+1.458e-03
+1.250e-03
+1.042e-03
+8.333e-04
+6.250e-04
+4.167e-04
+2.083e-04
+1.176e-17
Max: +3.231e-02
Elem: LOBULE2-1.7779
Node: 111181
Min: +1.176e-17
FLVEL
High Perm
FLVEL, Magnitude
(Avg: 75%)
+3.231e-02
+2.500e-03
+2.292e-03
+2.083e-03
+1.875e-03
+1.667e-03
+1.458e-03
+1.250e-03
+1.042e-03
+8.333e-04
+6.250e-04
+4.167e-04
+2.083e-04
+9.665e-19
Max: +3.231e-02
Elem: LOBULE2-1.7779
Node: 111181
Min: +9.665e-19
FLVEL
Low Perm
Fluid Velocity (um/s)
8000
6000
4000
2000
0
0
50
100
150
200
250
300
350
400
450
Path Length (um)
PV Medium Low Perm No Tumor
PV Medium Low Perm Tumor
PV Medium High Perm No Tumor
PV Medium High Perm Tumor
Control No Tumor Path
Control Tumor Path

(B)

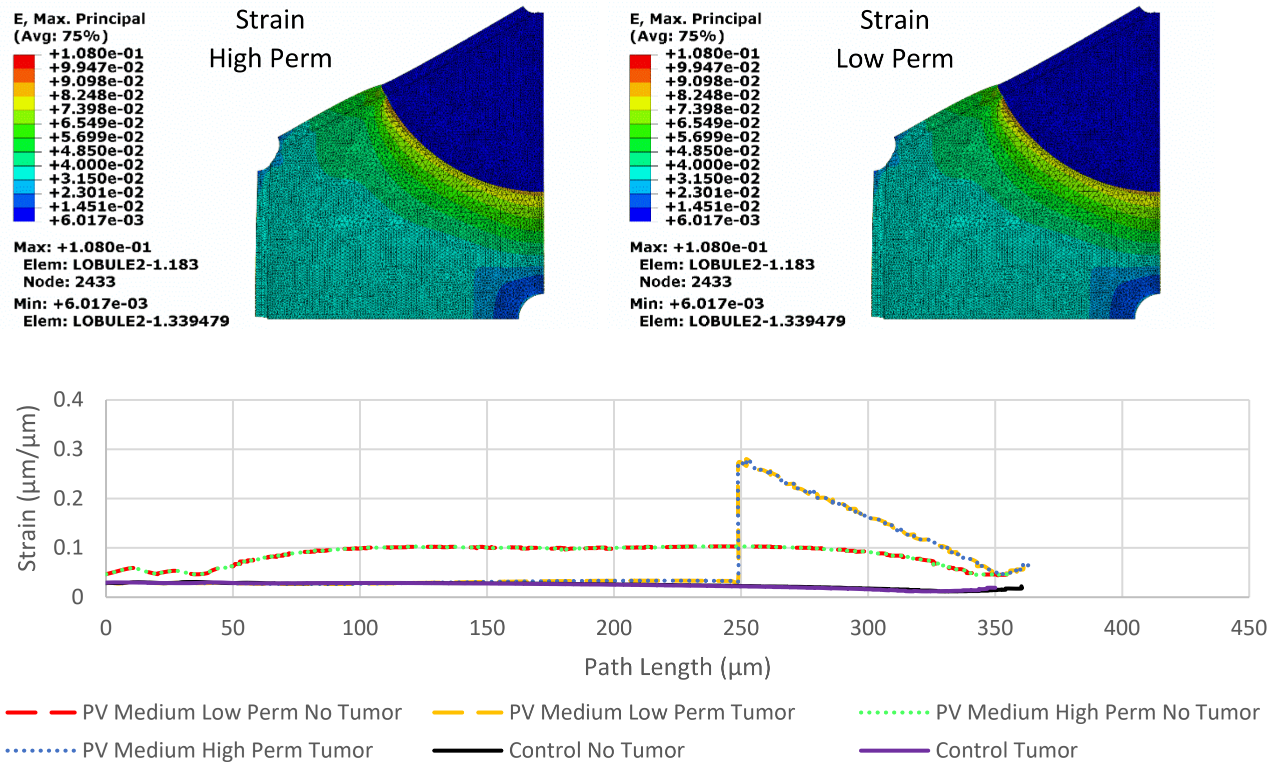
E, Max. Principal
(Avg: 75%)
+1.080e-01
+9.947e-02
+9.098e-02
+8.248e-02
+7.398e-02
+6.549e-02
+5.699e-02
+4.850e-02
+4.000e-02
+3.150e-02
+2.301e-02
+1.451e-02
+6.017e-03
Max: +1.080e-01
Elem: LOBULE2-1.183
Node: 2433
Min: +6.017e-03
Elem: LOBULE2-1.339479
Strain
High Perm
E, Max. Principal
(Avg: 75%)
+1.080e-01
+9.947e-02
+9.098e-02
+8.248e-02
+7.398e-02
+6.549e-02
+5.699e-02
+4.850e-02
+4.000e-02
+3.150e-02
+2.301e-02
+1.451e-02
+6.017e-03
Max: +1.080e-01
Elem: LOBULE2-1.183
Node: 2433
Min: +6.017e-03
Elem: LOBULE2-1.339479
Strain
Low Perm
Strain (µm/µm)
0.4
0.3
0.2
0.1
0
0
50
100
150
200
250
300
350
400
450
Path Length (µm)
PV Medium Low Perm No Tumor
PV Medium Low Perm Tumor
PV Medium High Perm No Tumor
PV Medium High Perm Tumor
Control No Tumor
Control Tumor

(C)

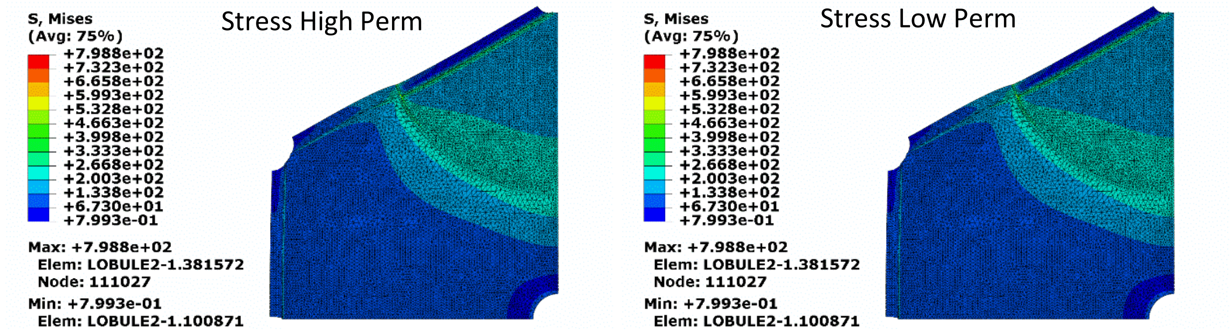

(D)

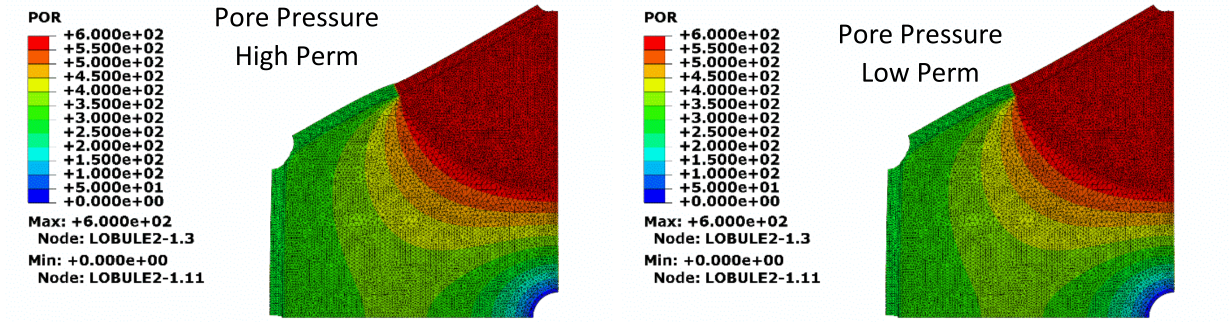

Figure S11. Images for the 400μm diameter tumor centered at the portal vein. Tumor was set as a 600 Pa pressure source. Images for (A) fluid velocity, (B) strain, (C) stress, and (D) pore pressure distribution throughout the quarter lobule. Graphs represent the fluid velocity and strain in the "tumor" and "no tumor" paths. Note the scale of fluid velocity and strain is much higher than other models.

(A)

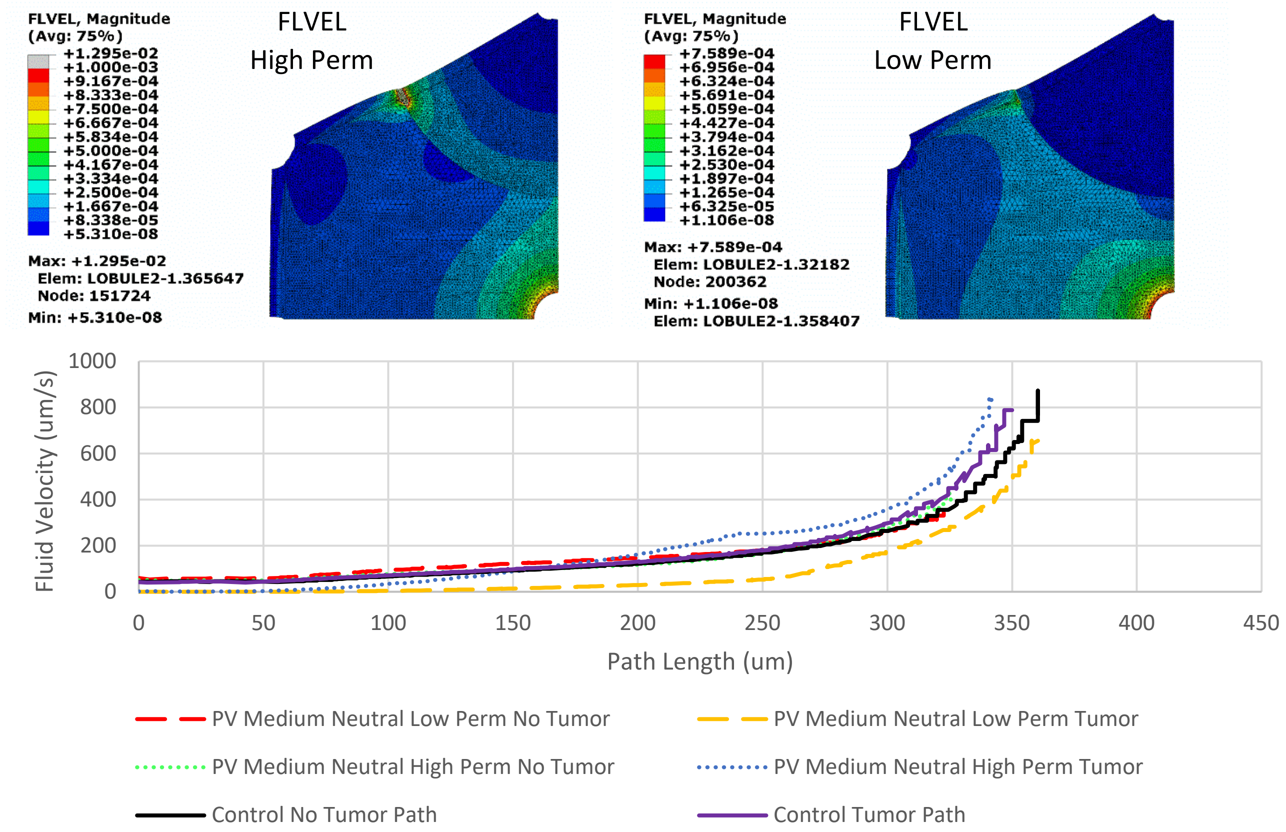
FLVEL, Magnitude
(Avg: 75%)
+1.295e-02
+1.000e-03
+9.167e-04
+8.333e-04
+7.500e-04
+6.667e-04
+5.834e-04
+5.000e-04
+4.167e-04
+3.334e-04
+2.500e-04
+1.667e-04
+8.338e-05
+5.310e-08
Max: +1.295e-02
Elem: LOBULE2-1.365647
Node: 151724
Min: +5.310e-08
FLVEL
High Perm
FLVEL, Magnitude
(Avg: 75%)
+7.589e-04
+6.956e-04
+6.324e-04
+5.691e-04
+5.059e-04
+4.427e-04
+3.794e-04
+3.162e-04
+2.530e-04
+1.897e-04
+1.265e-04
+6.325e-05
+1.106e-08
Max: +7.589e-04
Elem: LOBULE2-1.32182
Node: 200362
Min: +1.106e-08
Elem: LOBULE2-1.358407
FLVEL
Low Perm
Fluid Velocity (um/s)
1000
800
600
400
200
0
0
50
100
150
200
250
300
350
400
450
Path Length (um)
PV Medium Neutral Low Perm No Tumor
PV Medium Neutral Low Perm Tumor
PV Medium Neutral High Perm No Tumor
PV Medium Neutral High Perm Tumor
Control No Tumor Path
Control Tumor Path

(B)

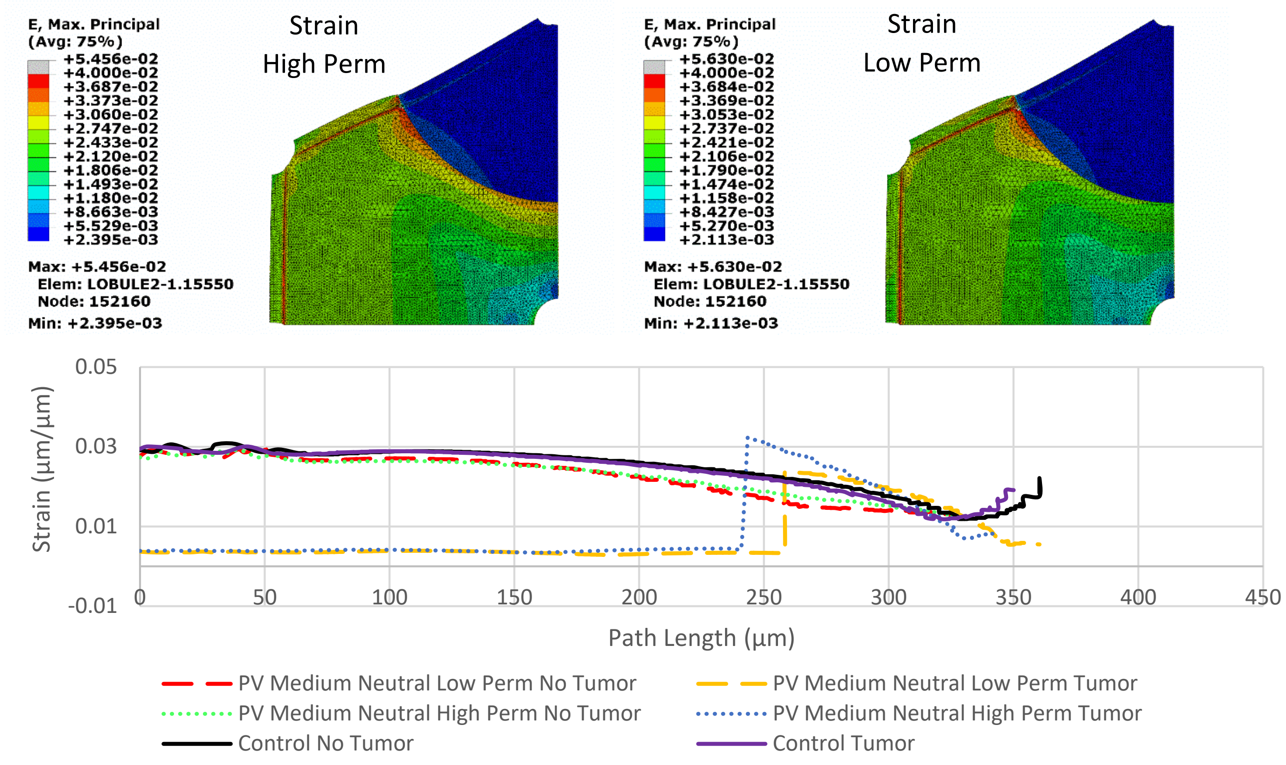
E, Max. Principal
(Avg: 75%)
+5.456e-02
+4.000e-02
+3.687e-02
+3.373e-02
+3.060e-02
+2.747e-02
+2.433e-02
+2.120e-02
+1.806e-02
+1.493e-02
+1.180e-02
+8.663e-03
+5.529e-03
+2.395e-03
Max: +5.456e-02
Elem: LOBULE2-1.15550
Node: 152160
Min: +2.395e-03
Strain
High Perm
E, Max. Principal
(Avg: 75%)
+5.630e-02
+4.000e-02
+3.684e-02
+3.369e-02
+3.053e-02
+2.737e-02
+2.421e-02
+2.106e-02
+1.790e-02
+1.474e-02
+1.158e-02
+8.427e-03
+5.270e-03
+2.113e-03
Max: +5.630e-02
Elem: LOBULE2-1.15550
Node: 152160
Min: +2.113e-03
Strain
Low Perm
Strain (µm/µm)
0.05
0.03
0.01
-0.01
0
50
100
150
200
250
300
350
400
450
Path Length (µm)
PV Medium Neutral Low Perm No Tumor
PV Medium Neutral Low Perm Tumor
PV Medium Neutral High Perm No Tumor
PV Medium Neutral High Perm Tumor
Control No Tumor
Control Tumor

C)

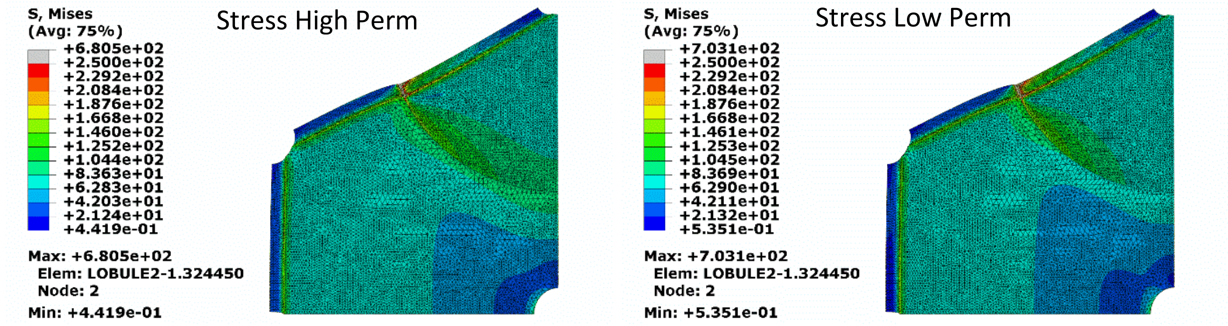

(D)

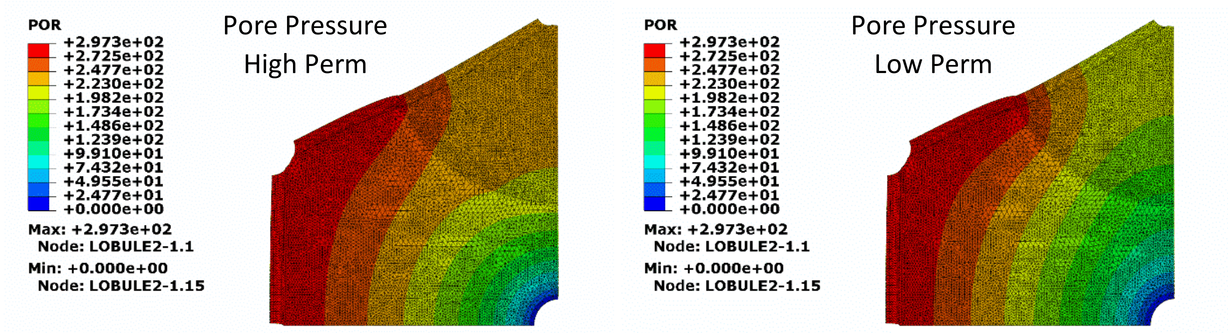

Figure S12. Images for the 400μm diameter tumor centered at the portal vein. Tumor was not set as a pressure source or sink. Images for (A) fluid velocity, (B) strain, (C) stress, and (D) pore pressure distribution throughout the quarter lobule. Graphs represent the fluid velocity and strain in the "tumor" and "no tumor" paths.

(A)

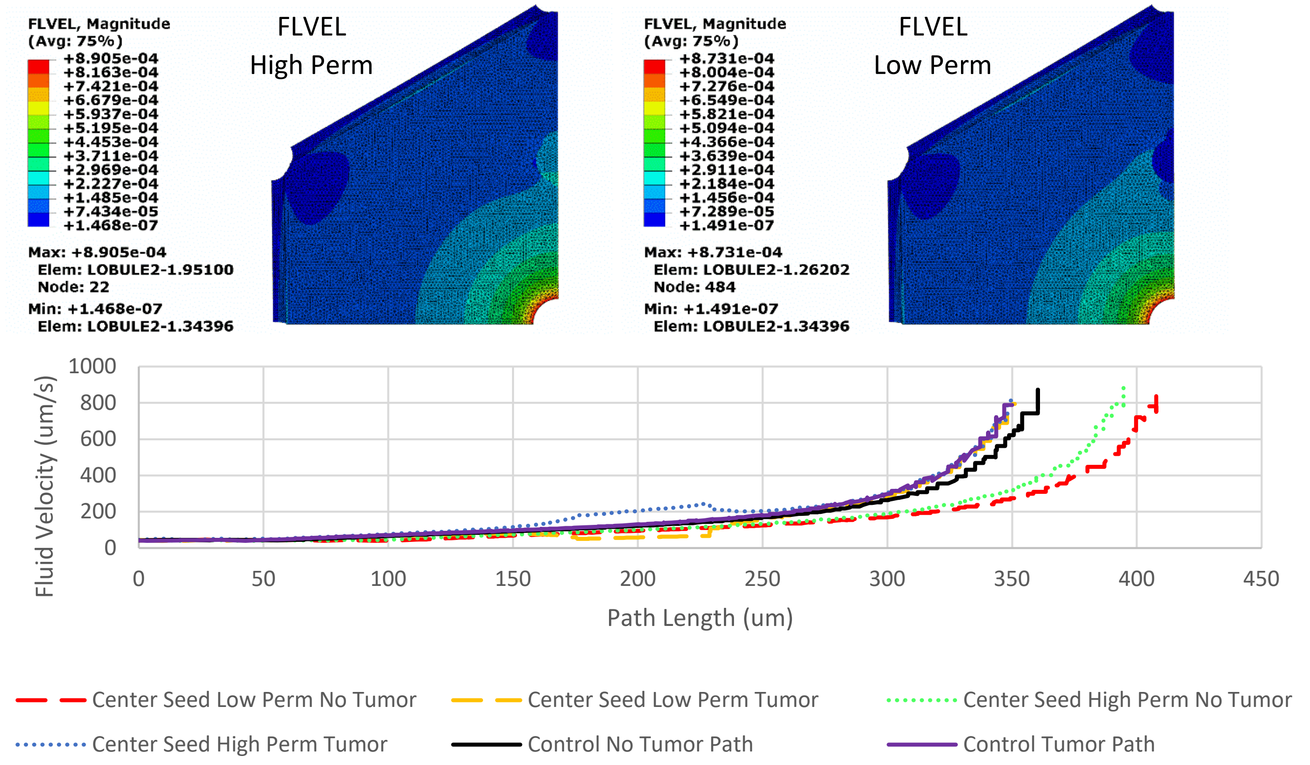
FLVEL, Magnitude
(Avg: 75%)
+8.905e-04
+8.163e-04
+7.421e-04
+6.679e-04
+5.937e-04
+5.195e-04
+4.453e-04
+3.711e-04
+2.969e-04
+2.227e-04
+1.485e-04
+7.434e-05
+1.468e-07
Max: +8.905e-04
Elem: LOBULE2-1.95100
Node: 22
Min: +1.468e-07
Elem: LOBULE2-1.34396
FLVEL
High Perm
FLVEL, Magnitude
(Avg: 75%)
+8.731e-04
+8.004e-04
+7.276e-04
+6.549e-04
+5.821e-04
+5.094e-04
+4.366e-04
+3.639e-04
+2.911e-04
+2.184e-04
+1.456e-04
+7.289e-05
+1.491e-07
Max: +8.731e-04
Elem: LOBULE2-1.26202
Node: 484
Min: +1.491e-07
Elem: LOBULE2-1.34396
FLVEL
Low Perm
Fluid Velocity (um/s)
1000
800
600
400
200
0
0
50
100
150
200
250
300
350
400
450
Path Length (um)
Center Seed Low Perm No Tumor
Center Seed Low Perm Tumor
Center Seed High Perm No Tumor
Center Seed High Perm Tumor
Control No Tumor Path
Control Tumor Path

(B)

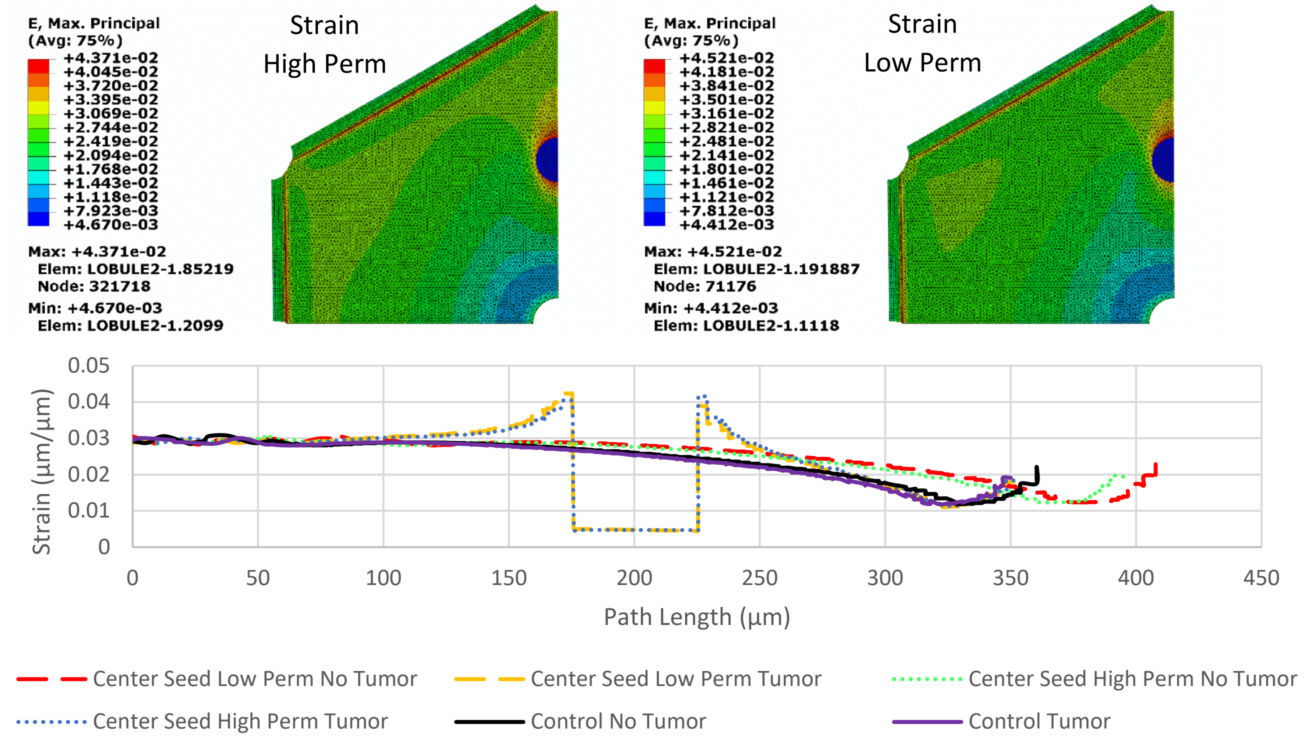
E, Max. Principal
(Avg: 75%)
+4.371e-02
+4.045e-02
+3.720e-02
+3.395e-02
+3.069e-02
+2.744e-02
+2.419e-02
+2.094e-02
+1.768e-02
+1.443e-02
+1.118e-02
+7.923e-03
+4.670e-03
Max: +4.371e-02
Elem: LOBULE2-1.85219
Node: 321718
Min: +4.670e-03
Elem: LOBULE2-1.2099
Strain
High Perm
E, Max. Principal
(Avg: 75%)
+4.521e-02
+4.181e-02
+3.841e-02
+3.501e-02
+3.161e-02
+2.821e-02
+2.481e-02
+2.141e-02
+1.801e-02
+1.461e-02
+1.121e-02
+7.812e-03
+4.412e-03
Max: +4.521e-02
Elem: LOBULE2-1.191887
Node: 71176
Min: +4.412e-03
Elem: LOBULE2-1.1118
Strain
Low Perm
Strain (µm/µm)
0.05
0.04
0.03
0.02
0.01
0
0
50
100
150
200
250
300
350
400
450
Path Length (µm)
Center Seed Low Perm No Tumor
Center Seed Low Perm Tumor
Center Seed High Perm No Tumor
Center Seed High Perm Tumor
Control No Tumor
Control Tumor

(C)

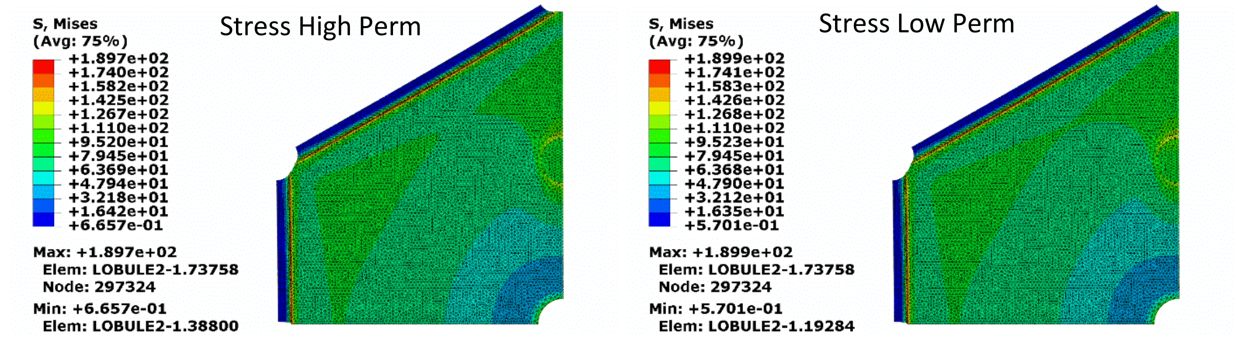

(D)

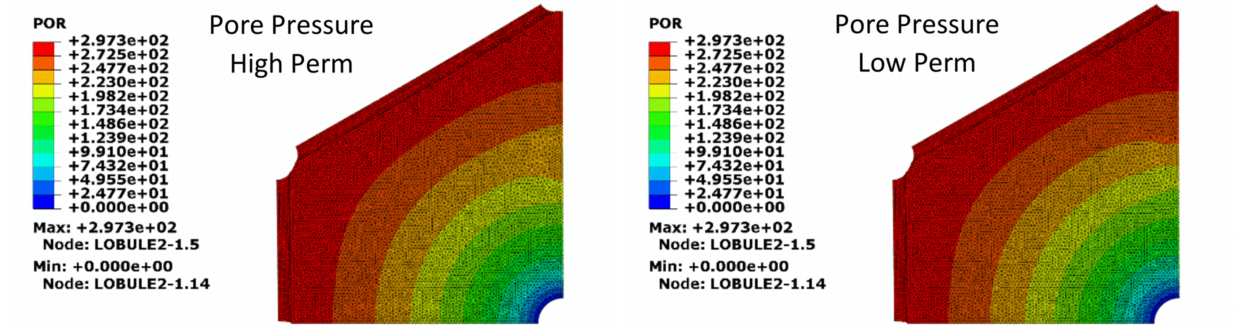

Figure S13. Images for the 50μm diameter tumor seed centered between the portal vein and central vein. Tumor was not set as a pressure source or sink. Images for (A) fluid velocity, (B) strain, (C) stress, and (D) pore pressure distribution throughout the quarter lobule. Graphs represent the fluid velocity and strain in the “tumor” and “no tumor” paths.

(A)

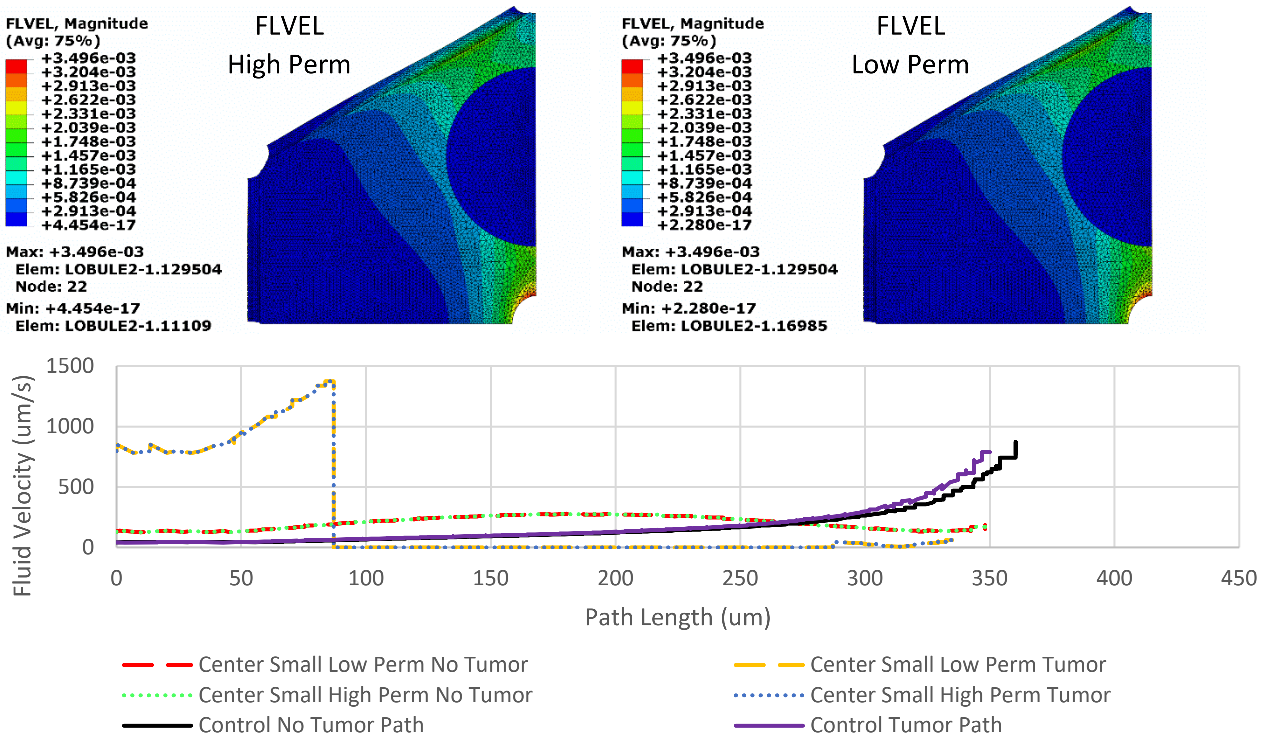

FLVEL, Magnitude
(Avg: 75%)
+3.496e-03
+3.204e-03
+2.913e-03
+2.622e-03
+2.331e-03
+2.039e-03
+1.748e-03
+1.457e-03
+1.165e-03
+8.739e-04
+5.826e-04
+2.913e-04
+4.454e-17
Max: +3.496e-03
Elem: LOBULE2-1.129504
Node: 22
Min: +4.454e-17
Elem: LOBULE2-1.11109
FLVEL
High Perm
FLVEL, Magnitude
(Avg: 75%)
+3.496e-03
+3.204e-03
+2.913e-03
+2.622e-03
+2.331e-03
+2.039e-03
+1.748e-03
+1.457e-03
+1.165e-03
+8.739e-04
+5.826e-04
+2.913e-04
+2.280e-17
Max: +3.496e-03
Elem: LOBULE2-1.129504
Node: 22
Min: +2.280e-17
Elem: LOBULE2-1.16985
FLVEL
Low Perm
Fluid Velocity (um/s)
1500
1000
500
0
0
50
100
150
200
250
300
350
400
450
Path Length (um)
Center Small Low Perm No Tumor
Center Small Low Perm Tumor
Center Small High Perm No Tumor
Center Small High Perm Tumor
Control No Tumor Path
Control Tumor Path

(B)

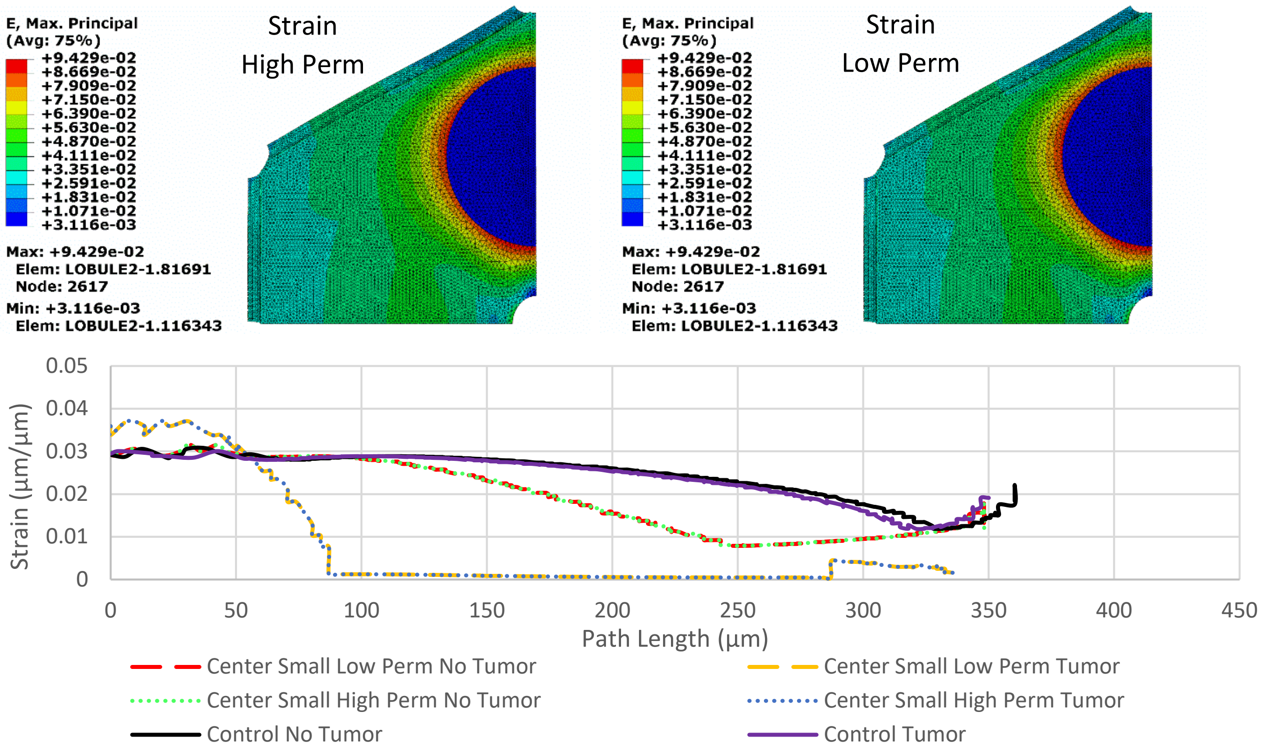

E, Max. Principal
(Avg: 75%)
+9.429e-02
+8.669e-02
+7.909e-02
+7.150e-02
+6.390e-02
+5.630e-02
+4.870e-02
+4.111e-02
+3.351e-02
+2.591e-02
+1.831e-02
+1.071e-02
+3.116e-03
Max: +9.429e-02
Elem: LOBULE2-1.81691
Node: 2617
Min: +3.116e-03
Elem: LOBULE2-1.116343
Strain
High Perm
E, Max. Principal
(Avg: 75%)
+9.429e-02
+8.669e-02
+7.909e-02
+7.150e-02
+6.390e-02
+5.630e-02
+4.870e-02
+4.111e-02
+3.351e-02
+2.591e-02
+1.831e-02
+1.071e-02
+3.116e-03
Max: +9.429e-02
Elem: LOBULE2-1.81691
Node: 2617
Min: +3.116e-03
Elem: LOBULE2-1.116343
Strain
Low Perm
Strain (µm/µm)
0.05
0.04
0.03
0.02
0.01
0
0
50
100
150
200
250
300
350
400
450
Path Length (µm)
Center Small Low Perm No Tumor
Center Small Low Perm Tumor
Center Small High Perm No Tumor
Center Small High Perm Tumor
Control No Tumor
Control Tumor

(C)

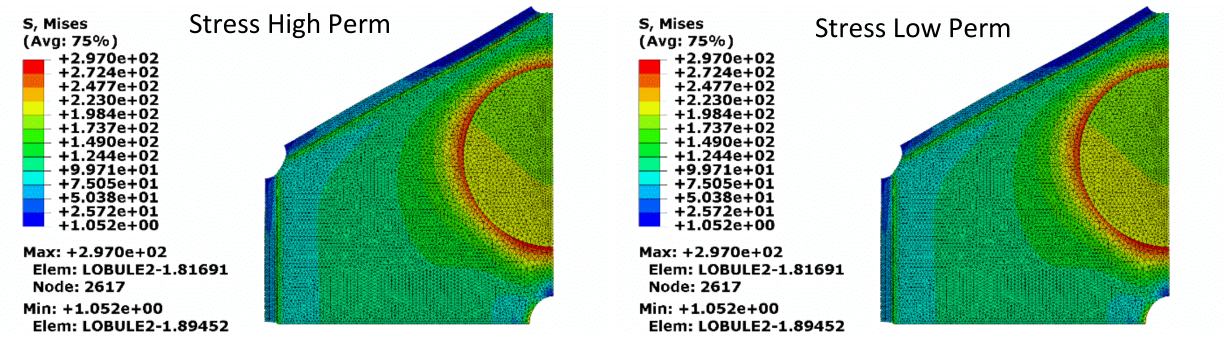

(D)

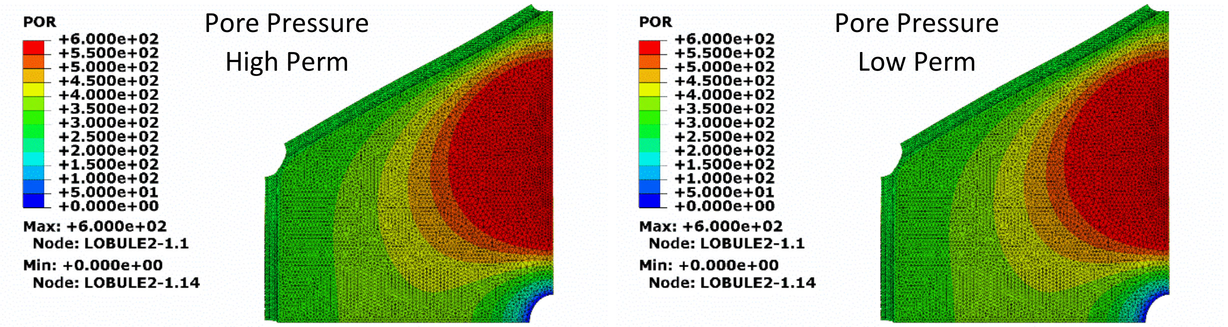

Figure S14. Images for the 200µm diameter tumor seed centered between the portal vein and central vein. Tumor was set as a pressure sink. Images for (A) fluid velocity, (B) strain, (C) stress, and (D) pore pressure distribution throughout the quarter lobule. Graphs represent the fluid velocity and strain in the "tumor" and "no tumor" paths. Note the scale of fluid velocity is higher than other models.

(A)

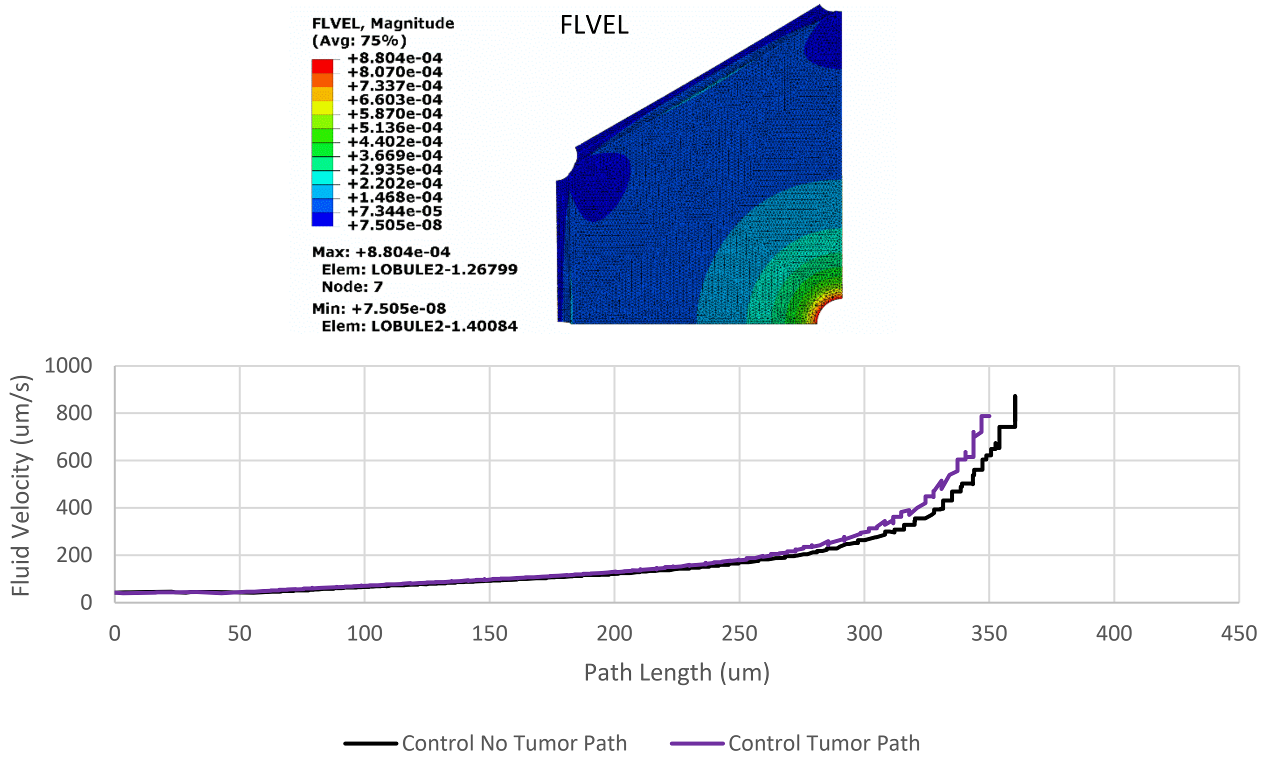
FLVEL, Magnitude
(Avg: 75%)
+8.804e-04
+8.070e-04
+7.337e-04
+6.603e-04
+5.870e-04
+5.136e-04
+4.402e-04
+3.669e-04
+2.935e-04
+2.202e-04
+1.468e-04
+7.344e-05
+7.505e-08
Max: +8.804e-04
Elem: LOBULE2-1.26799
Node: 7
Min: +7.505e-08
Elem: LOBULE2-1.40084
FLVEL
Fluid Velocity (um/s)
1000
800
600
400
200
0
0
50
100
150
200
250
300
350
400
450
Path Length (um)
Control No Tumor Path
Control Tumor Path

(B)

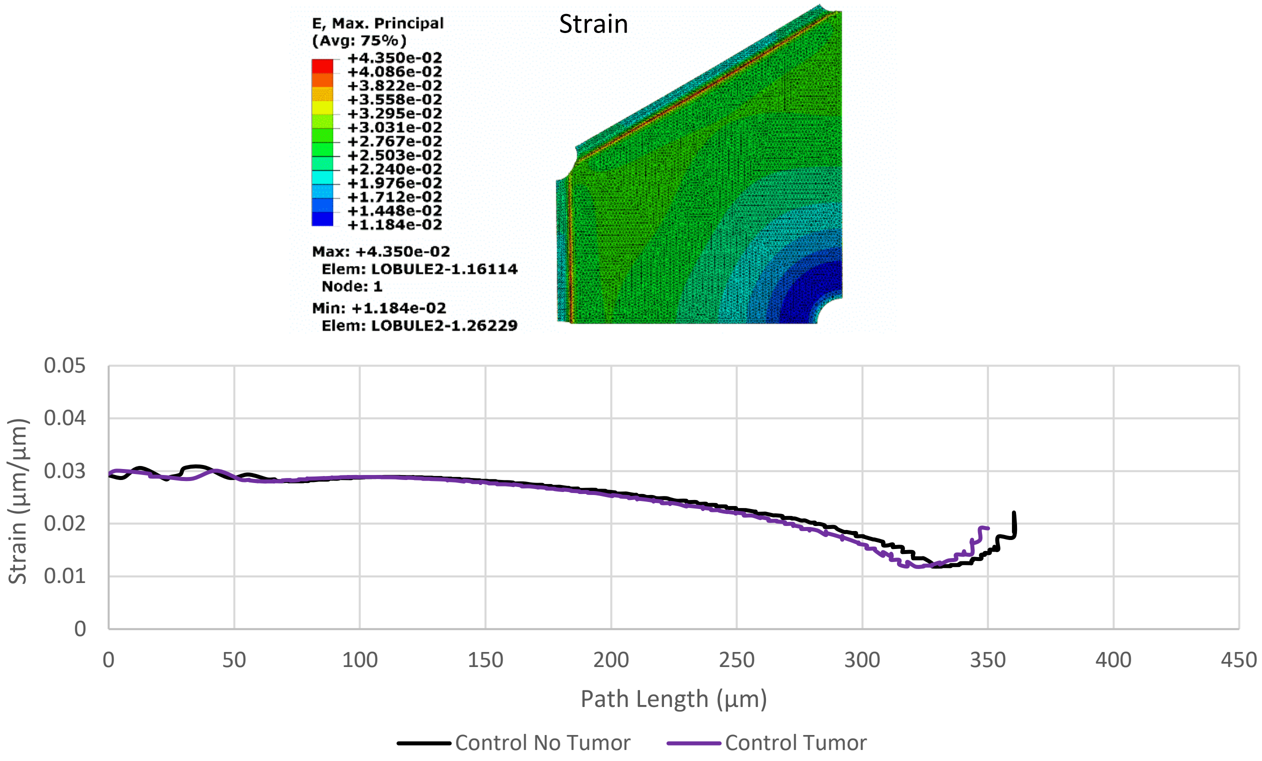
E, Max. Principal
(Avg: 75%)
+4.350e-02
+4.086e-02
+3.822e-02
+3.558e-02
+3.295e-02
+3.031e-02
+2.767e-02
+2.503e-02
+2.240e-02
+1.976e-02
+1.712e-02
+1.448e-02
+1.184e-02
Max: +4.350e-02
Elem: LOBULE2-1.16114
Node: 1
Min: +1.184e-02
Elem: LOBULE2-1.26229
Strain
Strain (µm/µm)
0.05
0.04
0.03
0.02
0.01
0
0
50
100
150
200
250
300
350
400
450
Path Length (µm)
Control No Tumor
Control Tumor

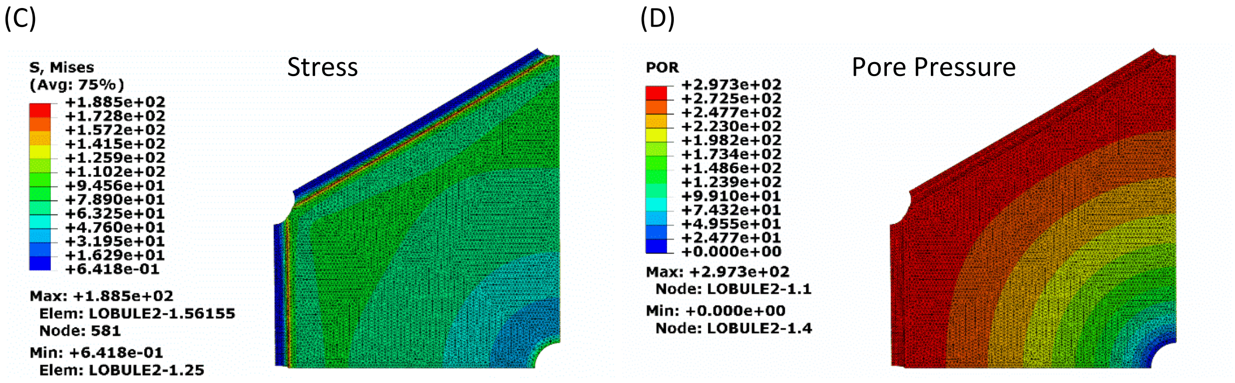

Figure S15. Images for the control lobule (there is no tumor anywhere in this model). Images for (A) fluid velocity, (B) strain, (C) stress, and (D) pore pressure distribution throughout the quarter lobule. Graphs represent the fluid velocity and strain in the "tumor" and "no tumor" paths (to keep path notation consistent with plots above). Data for control fluid velocity and strain can be found in other model graphs, but is also displayed here for clarity.

Supplemental Materials Bibliography

Bonfiglio A, Leungchavaphongse K, Repetto R, Siggers JH (2010) Mathematical Modeling of the Circulation in the Liver Lobule. J Biomech Eng 132:111011–111011. doi: 10.1115/1.4002563

Evans DW, Moran EC, Baptista PM, et al (2013) Scale-dependent mechanical properties of native and decellularized liver tissue. Biomech Model Mechanobiol 12:569–580. doi: 10.1007/s10237-012-0426-3

Lu Q, Ling W, Lu C, et al (2015) Hepatocellular Carcinoma: Stiffness Value and Ratio to Discriminate Malignant from Benign Focal Liver Lesions. Radiology 275:880–888. doi: 10.1148/radiol.14131164

Netti PA, Berk DA, Swartz MA, et al (2000) Role of Extracellular Matrix Assembly in Interstitial Transport in Solid Tumors. Cancer Res 60:2497–2503.

Nishii K, Reese G, Moran EC, Sparks JL (2016) Multiscale computational model of fluid flow and matrix deformation in decellularized liver. J Mech Behav Biomed Mater 57:201–214. doi: 10.1016/j.jmbbm.2015.11.033

Pishko GL, Astary GW, Mareci TH, Sarntinoranont M (2011) Sensitivity Analysis of an Image-Based Solid Tumor Computational Model with Heterogeneous Vasculature and Porosity. Ann Biomed Eng 39:2360–2373. doi: 10.1007/s10439-011-0349-7

Swabb EA, Wei J, Gullino PM (1974) Diffusion and Convection in Normal and Neoplastic Tissues. Cancer Res 34:2814–2822.

Venkatesh SK, Yin M, Glockner JF, et al (2008) Magnetic Resonance Elastography of Liver Tumors- Preliminary Results. AJR Am J Roentgenol 190:1534–1540. doi: 10.2214/AJR.07.3123
